# Robust point cloud lightweighting with multi-scale adaptive filtering and entropy-driven subdivision

**DOI:** 10.1371/journal.pone.0353953

**Published:** 2026-07-24

**Authors:** Weibo Zeng, Xinyu Gao, Qi Lu, Ning Zhu, Mengchan Li, Wenjing Cai

**Affiliations:** 1 Anhui Provincial Key Laboratory of Realistic Geographic Environment, Chuzhou, China; 2 School of Geographic Engineering and Spatial Information, Chuzhou University, Chuzhou, China; 3 School of Resources and Environmental Engineering, Anhui University, Hefei, China; University of Oxford, UNITED KINGDOM OF GREAT BRITAIN AND NORTHERN IRELAND

## Abstract

Current point cloud simplification methods for complex ground objects face a persistent challenge: balancing noise robustness and geometric detail preservation. To resolve this, we present a lightweight simplification framework that integrates multi-scale adaptive filtering, entropy-driven spatial partitioning, and an enhanced medial axis transform (MAT). This framework incorporates three targeted technical innovations: (1) An adaptive sliding window polynomial fitting filter with multi-resolution weight adjustment, which achieves coordinated noise suppression and sharp feature preservation; (2) A curvature-weighted enhanced MAT algorithm that reduces skeletal artifacts and topological fractures; (3) An entropy-driven adaptive recursive axis-aligned bounding box (AABB) partitioning strategy, which mitigates the inherent trade-off of conventional uniform partitioning: memory waste in sparse regions and feature loss in dense areas. We validated this framework using self-collected datasets of buildings, vegetation, and roads, and further verified its generalization performance on the public STPLS3D benchmark. Our method achieves an average noise removal rate of 87.76%, representing an average improvement of 11.99% over the baseline method; edge retention is 83.3%, an average improvement of 7.35%; the topological integrity and branch accuracy of the skeleton extraction reached 0.93 and 0.95, respectively, both of which were the best among the tested algorithms; the mean error in normal estimation was as low as 3.34%, and the average point-to-surface distance and fracture rate in 3D reconstruction were the lowest. This method provides a point cloud processing solution that combines accuracy and efficiency for fields such as 3D geographic information modeling and scene reconstruction.

## 1. Introduction

Efficient representation and simplification of terrestrial point clouds are foundational tasks in 3D geographic information modeling, urban planning, and environmental monitoring. This work requires balancing three often conflicting objectives: high-precision geometric feature extraction, robust morphological optimization in complex scenes, and computationally efficient normal estimation. Reconciling these objectives is critical to ensuring the accuracy, efficiency, and reliability of subsequent 3D modeling and analysis workflows. Point cloud datasets are frequently degraded by three key artifacts: noise interference [1], non-uniform density distribution [2], and blurred geometric details [3]. These artifacts markedly reduce the accuracy of semantic segmentation [4] and the efficiency of 3D reconstruction [5] in complex scenes. Traditional skeleton extraction methods face a fundamental limitation: they either incur excessive computational costs or exhibit high noise sensitivity, making it difficult to balance detail preservation and noise robustness [6]. Normal estimation techniques, meanwhile, have notable shortcomings in handling sharp geometric features [7] and suppressing outlier points [8]. Existing simplification frameworks have made meaningful advances in geometric feature manipulation [9] and morphological refinement [10], but most lack dynamic coupling and inter-module information exchange mechanisms [11]. This deficiency impairs topological consistency during hierarchical feature aggregation [12], while noise interference often creates jagged semantic boundaries [13] that hinder the collaborative optimization of multi-scale ground object structures [14]. Multi-scale feature fusion and adaptive optimization strategies [15] are therefore essential to address these challenges, particularly for improving noise robustness [16], detail integrity [17], and computational efficiency [18] when processing point clouds of complex ground objects.

Existing research on point cloud skeleton extraction can be broadly divided into three categories: distance-based methods [19], geometric feature-based methods [20], and spatial partitioning-based methods [21], with notable algorithmic advances made in recent years [22]. Distance-based methods are the foundational approach, and offer relatively high efficiency for simple topological structures [23]. These methods identify medial axis features by constructing a distance field function [24]. Early studies identified skeleton points by calculating the radius of the maximum inscribed sphere [25], an approach that achieves high accuracy for regular shapes such as pipes and cylinders [26]. Subsequent improvements, such as anisotropic distance transform combined with principal component analysis [27], greatly enhanced branch recognition performance [28]. Li [29] later proposed a multi-scale distance fusion strategy that builds hierarchical distance fields, mitigating issues caused by point cloud density variation. The performance of these methods, however, is heavily dependent on point cloud quality. Random noise can distort the distance field gradient, causing skeleton point shifts or false branch generation [30]. Non-uniform sampling, in turn, reduces the accuracy of distance field calculations, leading to lower spatial resolution and skeletal discontinuities [31]. These issues are particularly pronounced when processing complex objects with high-frequency details, such as gaps between leaves or interwoven branch structures, where distance field fluctuations produce non-physical variations in the skeleton path. Geometric feature methods, such as those based on curvature extremal points [32], deliver high representation accuracy for models with prominent geometric features [33]. Enhanced normal vector clustering algorithms, which integrate geodesic distance constraints and divergence threshold control, can effectively preserve the topological integrity of building point clouds. To address the limitations of purely geometric feature-driven methods, spatial partitioning approaches provide complementary solutions through global path optimization. These methods refine extracted skeletal key points, ultimately producing semantically accurate segmentation results with clear boundaries. They can also construct efficient, realistic dynamic paths for ground object skeletons, based on the global minimum-cost path from each point to the root node [34]. Our synthesis of existing skeleton extraction research identifies three core unresolved challenges. First, excessive reliance on geometric features reduces the cross-scene generalizability of existing methods. Second, high computational efficiency often comes at the cost of fine detail preservation. Third, it remains difficult to achieve both high accuracy and computational efficiency when processing complex topological structures.

The dominant algorithms for point cloud normal estimation fall into four categories: plane fitting-based methods [35], principal component analysis (PCA)-based algorithms [36], Voronoi-based algorithms [37,38], and deep learning-based approaches [39,40]. L. et al. [41] demonstrated that local plane fitting-based methods provide a simple, generally effective solution for normal estimation, but lack high accuracy and can produce significant computational errors, especially for complex or noisy models. A multi-scale neighborhood translation strategy [42] addresses this limitation by dynamically adjusting the neighborhood scale, effectively suppressing both noise-induced errors and biases from sharp features. Moving Least Squares (MLS) surface fitting [43] further constructs a more robust differential geometry model of the surface, preserving detailed feature accuracy while resolving discontinuous normal propagation within neighborhoods. Overall, local plane fitting methods have relatively low computational complexity, but limited adaptability to complex shapes and high susceptibility to outliers. PCA-based algorithms are simple and easy to implement, but sensitive to variations in the size of the local point set, resulting in poor stability. Multi-scale PCA methods [44] can achieve relatively accurate normal estimation for point clouds with varying levels of detail, by adaptively incorporating different spatial scales. Voronoi-based algorithms, originally developed for noise-free point clouds, represent another key approach. Combining the strengths of Voronoi-based methods [45] with PCA principles can partially mitigate the impact of point cloud noise on normal estimation. Researchers have also leveraged the strong coupling between globally consistent normals and the canonical winding number field to improve the orientation consistency of noisy point clouds [46], boosting overall estimation accuracy. Advances in deep learning theory, alongside improvements in computer hardware (e.g., GPUs and CPUs) and frameworks (e.g., TensorFlow, Caffe, and PyTorch), have enabled new, more efficient methods for normal estimation. These methods further reduce the impact of noise on normal estimation and improve robustness. For example, projecting Hough space onto a trained convolutional neural network (CNN) structure [47] enhances robustness against outlier anomalies and data acquisition noise, enabling better adaptation to point density fluctuations. Using a deep neural network (DNN) to map normal vectors at sharp feature areas, however, has a key limitation: the resulting estimates fail to capture the actual discontinuous changes in normals. The SharpNet framework [48], by comparison, uses a feature classification paradigm to reformulate the normal estimation problem. This approach preserves the discrete distribution of sharp features while eliminating the quantization errors inherent in traditional discretization methods, offering a new direction for normal estimation in complex geometries.

In summary, despite substantial advances in point cloud filtering, skeleton extraction, and normal estimation, critical challenges remain unaddressed. A core unresolved issue is the elimination of non-uniform noise without compromising local differential structures, a limitation that prevents accurate discrimination between noise points and fine-grained feature points during simplification. While geometric feature analysis can partially offset the weak noise resistance of distance-based methods, their computational efficiency and detail preservation require systematic improvement via structured optimization strategies such as spatial partitioning. For large-scale point clouds, conventional uniform partitioning fails to account for density variations, resulting in inefficient memory usage in sparse regions and irreversible feature loss in dense areas. Deep learning-centric point cloud processing frameworks, by contrast, rely heavily on large-scale, high-quality annotated training datasets. In practical engineering scenarios involving complex urban ground objects, few-shot or unannotated data are the norm. In these settings, such frameworks exhibit severe degradation in generalization performance and are highly prone to overfitting. They also demand extensive GPU memory and computational resources for inference, and are further limited by complex parameter tuning workflows and poor model interpretability. These barriers prevent their practical deployment in engineering scenarios with constrained computing power, such as field operations and mobile terminals. Existing hybrid frameworks alleviate these limitations to some degree, but retain inherent flaws: insufficient coupling between geometric constraints and network optimization, and excessive parameter tuning complexity. To resolve these research gaps, we propose a novel point cloud simplification method based on multi-scale adaptive filtering and entropy-driven partitioning. Our key technical contributions are outlined below:

We design a novel adaptive sliding window polynomial fitting filter to suppress noise interference while preserving key details and features of the point cloud’s local geometric structure. This filter integrates a multi-resolution weight adjustment mechanism and a two-level window optimization strategy for feature edges, delivering high-fidelity point cloud data that provides a robust foundation for subsequent normal estimation and 3D reconstruction.

We develop an enhanced medial axis transform (MAT) algorithm that incorporates curvature-weighted sampling and dynamic radius neighborhood search. These innovations optimize skeleton point density and enable accurate identification of distance field local maxima, ensuring the integrity of geometric features while reducing false branches and topological discontinuities.

We propose a hierarchical adaptive recursive spatial partitioning algorithm based on axis-aligned bounding boxes (AABBs) to overcome the limitations of conventional uniform partitioning, including poor memory utilization in sparse regions and severe feature loss in dense areas. This algorithm constructs a dynamic segmentation threshold based on the weight entropy of sub-bounding boxes, enabling adaptive division of multi-scale spatial constraint domains and efficient structured reorganization of point cloud distributions.

The remainder of this paper is organized as follows: Section 2 provides a comprehensive overview of our methodological approach and data sources. Section 3 presents our experimental results and detailed quantitative analysis. Section 4 summarizes the key findings of this study.

## 2. Method

**Fig 1:**
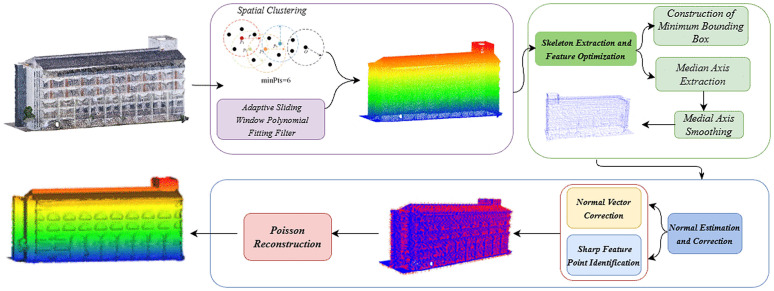
General workflow of the proposed method.

In this work, we propose a robust simplification method for mixed urban point cloud models. This approach integrates multi-scale adaptive filtering and entropy-driven partitioning to achieve geometry-preserving reconstruction and topology-consistent lightweight representation of skeleton features from complex urban ground objects. The workflow of our method consists of three core steps. First, we apply an adaptive sliding window polynomial fitting filter to suppress noise while preserving key geometric features. Second, we use hierarchical bounding boxes to delineate spatial boundaries and identify extreme values, coupled with a medial axis transform to capture the object skeleton and enhance topological consistency. Finally, we screen feature points for subsequent normal vector correction, generating lightweight, low-noise point cloud data optimized for 3D reconstruction. The overall workflow is illustrated in Fig 1.

### 2.1. Point Cloud Sampling and Filtering

#### 2.1.1. Density-Based Spatial Clustering Algorithm.

(1) Density Neighborhood Definition and Core Point Determination

The density-based spatial clustering algorithm (DBSCAN) [49] groups density-connected data points into the same cluster, while identifying isolated noise points. We first initialize the algorithm parameters, including the neighborhood search radius. We then calculate the neighborhood of each point and identify core points.

For an arbitrary point $p_{i}$, we define its ε-neighborhood as:


$$\begin{array}{c} N_{\epsilon }\left(p_{i}\right)=\{p_{j}\in P|\|p_{i}-p_{j}\|\leq \epsilon \} \end{array}$$ (1)

In Eq. (1), $N_{\epsilon }\left(p_{i}\right)$ is the set of points within a Euclidean distance ϵ from point$\;p_{i}$, $\left\|p_{i}-p_{j}\right\|$ denotes the Euclidean distance between $p_{i}$ and $p_{j}$, and ϵ is the neighborhood search radius.

We count the number of points in the neighborhood $\left|N_{\epsilon }\left(p_{i}\right)\right|$, and identify core points using a preset threshold (MinPts). If:


$$\begin{array}{c} \left|N_{\epsilon }\left(p_{i}\right)\right|\geq MinPts \end{array}$$ (2)

$p_{i}$is marked as a core point; otherwise, it is marked as a boundary point or a noise point. Boundary and noise points are then processed using the density-based clustering method.

(2) Cluster Expansion and Lightweight Strategy

We first select a core point $p_{i}$ to initialize cluster expansion, and recursively absorb neighboring points through density reachability to form the initial cluster $C_{k}$. For each generated cluster $C_{k}$, we calculate the geometric centroid of all its points as $\overset{\text{light}}{\underset{k}{C}}$, using Eq. (3). This compresses the original high-density point set $C_{k}$ into a single centroid $\overset{\text{light}}{\underset{k}{C}}$, replacing the redundant original point set.


$$\begin{array}{c} \overset{\text{light}}{\underset{k}{C}}=\left\{\frac{\text{1}}{\left|C_{k}\right|}\sum_{p\in C_{k}}p\right\} \end{array}$$ (3)

#### 2.1.2. Adaptive Sliding Window Polynomial Fitting Filter.

**Fig 2:**
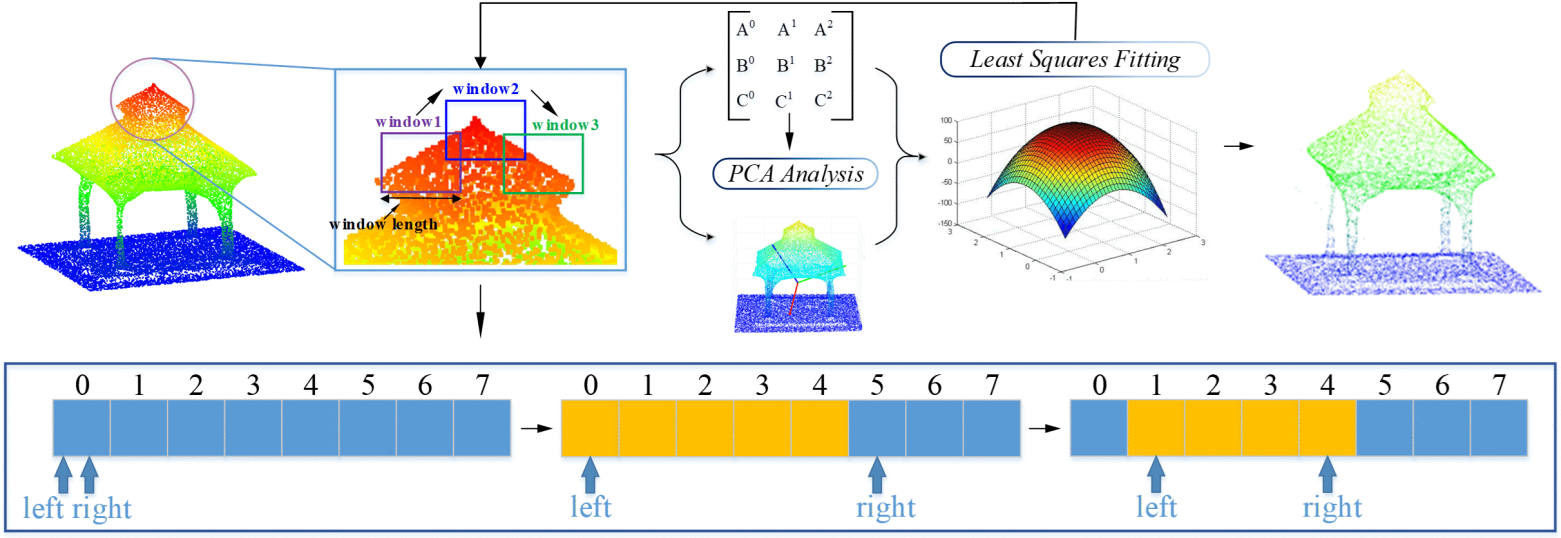
Schematic diagram of sliding window points cloud denoising process.

Complex geometric features (such as sharp edges and high-frequency noise) make it difficult to suppress noise effectively while maintaining geometric feature integrity. We therefore designed an adaptive sliding window polynomial fitting filtering algorithm, which retains critical details of the local geometric structure and feature distribution of point clouds during noise suppression. The principle of the algorithm is shown in Fig 2.

(1) Determination of Window Width and Polynomial Order

We define the sliding window size L and polynomial order p, which is subject to the constraint in Eq. (4):


$$\begin{array}{c} L>p+\text{1} \end{array}$$ (4)

We determine the value of L using the empirical formula L=[2p+3], derived from comparative experiments on point clouds of three typical ground objects: buildings, vegetation, and roads. We set the polynomial order p to three levels widely used in practical applications: 1 for linear fitting, 2 for quadratic fitting, and 3 for cubic fitting. For each order p, we specify seven candidate window sizes L: p+2,p+3,p+4,p+5,2p+3,2p+4, and 2p+5. We perform quantitative evaluation using three core metrics: noise removal rate, edge preservation, and fitting error. The results show that overall performance is optimal when L=[2p+3].

(2) Construction of the Data Matrix

We set the window width to W=2M+1 and the polynomial order to P=N (where N<2M+1). For each point $X^{\left[N\right]}$ in the point cloud, we construct a data window containing W points with $X_{k}$ as the window center. We then build a data matrix A with W rows and N+1 columns. The element in the i−th row and j−th column of matrix A is $\overset{j}{\underset{\left[i\right]}{X}}$, the j−th power of each point in the window. We perform PCA on the window center point $X_{i}$ following Eq. (5):


$$\begin{array}{c} Cov=\frac{\text{1}}{L}\sum_{i=k-M}^{K+M}\left(P_{i}-\mu \right){\left(P_{i}-\mu \right)}^{T} \end{array}$$ (5)

In Eq. (5), Cov denotes the covariance matrix, L is the number of points in the window, K is the index of the window center point $X_{k}$, M is the window half-width parameter, $P_{i}$ is the feature vector of the i−th point in the window, μ is the mean value of the point set ${\{P}_{i}\}$ in the window, and $\left(P_{i}-\mu \right)$ is the de-meaned vector.

We use the first two principal component vectors u and v to construct a tangent plane, and project neighborhood points to the local coordinate system. This eliminates the interference of normal dimension noise on the polynomial, as shown in Eq. (6):


$$\begin{array}{c} \left(u_{i},v_{i}\right)=\left(\left(P_{i}-\mu \right)\cdot u,\left(P_{i}-\mu \right)\cdot v\right) \end{array}$$ (6)

(3) Minimization of Fitting Error

We solve the least squares solution of the linear system A*θ=Y, where Y is the vector of data points in the window. Solving for θ requires multiplying both sides of the equation by the inverse of A, which may not exist for all datasets. We therefore use the Moore-Penrose pseudo-inverse of A instead, as shown in Eq. (7). By calculating the pseudo-inverse $A^{+}$ of A, we achieve optimal separation of noise distribution and geometric features. The polynomial coefficients are calculated using Eq. (8):


$$\begin{array}{c} A^{+}={\left(A^{T}\cdot A\right)}^{-\text{1}}{\cdot A}^{T} \end{array}$$ (7)

$$\begin{array}{c} \theta =A^{+}\cdot Y \end{array}$$ (8)

(4) Calculation of Filtered Value for Window Center Point

Using the obtained polynomial coefficient θ, we calculate the filtered value of the window center point $X_{k}$, as shown in Eq. (9):


$$\begin{array}{c} Ysmooth[n]=\theta_{\text{0}}+\theta_{\text{1}}\cdot X[n]+\theta_{\text{2}}\cdot X[n{]}^{\text{2}}\ldots \end{array}$$ (9)

Here, Ysmooth[n] denotes the filtered value, θ is the polynomial fitting coefficient, and X[n] is the position coordinate of the data point in the window relative to the center point.

We introduce a multi-resolution processing mechanism, which uses weighted coefficients to dynamically adjust filtering results across scales. This enables effective complementarity between coarse-scale noise suppression and fine-scale feature preservation. The coarse-scale weight, defined in Eq. (10), quantifies the smoothness within the coarse-scale window to enhance noise reduction. The fine-scale weight, defined in Eq. (11), measures the feature salience within the fine-scale window to retain sharp edges and texture details.


$$\begin{array}{c} W_{coarse}=\frac{\text{1}}{\overset{\text{2}}{\underset{local}{\sigma }}+\in } \end{array}$$ (10)

In Eq. (10), $W_{coarse}$ denotes the coarse-scale weight, $\sigma_{local}$ is the data variance in the coarse-scale window, and ∈ is an extremely small constant used to prevent the denominator from being zero.


$$\begin{array}{c} W_{fine}=\frac{\left||\nabla P|\right|}{{\left||\nabla P|\right|}_{max}} \end{array}$$ (11)

In Eq. (11), $W_{fine}$ denotes the fine-scale weight, and ∇P is the gradient magnitude in the fine-scale window.

We adopt a two-level window strategy in feature edge areas to dynamically optimize filtering parameters, balancing noise suppression and feature preservation, as shown in Eq. (12):


$$\begin{array}{c} y_{final}=\alpha yL_{\text{1}}+(\text{1}-\alpha )yL_{\text{2}} \end{array}$$ (12)

Here, $L_{\text{1}}$ denotes the primary window width, and $L_{\text{2}}$ denotes the secondary window width. For sharp feature regions such as building edges and road boundaries, the primary window width is fixed at $L_{\text{1}}=\text{2}M+\text{1}$, where M is the window half-width parameter. Three candidate values are specified for the secondary window width $L_{\text{2}}$: M, M+1, and M+2. The fusion weight α is set to a value range of [0.4, 0.9] with a step size of 0.1, giving a total of six candidate values. Comprehensive evaluation is performed using four metrics: noise removal rate (30% weight), edge retention (35% weight), normal consistency (25% weight), and computational efficiency (10% weight). After testing and analysis of the sharp feature regions across the three typical ground objects, the optimal parameter configuration is finalized as $L_{\text{1}}=\text{2}M+\text{1}$, $L_{\text{2}}=M+\text{1}$, with α∈[0.6,0.8].

(5) Window Sliding and Iteration

We slide the window by one point along the point sequence, and repeat the operations from Step (2) to Step (4) until the window traverses the entire point cloud. This ensures every point in the dataset is processed exactly once as the window center.

### 2.2. Skeleton Extraction and Feature Optimisation

Our proposed point cloud simplification method integrates medial axis constraints and morphological optimization, and achieves coordinated optimization of topological consistency and computational efficiency via a multi-level coupling mechanism. We leverage AABB for rapid localization of the point cloud’s spatial extreme boundaries, and integrate a medial axis transform algorithm to extract topology-preserving skeletons through local maximum detection of the distance field [50]. We also adopt a dual-constraint criterion combining spatial proximity and normal consistency to generate a robust medial axis network.

#### 2.2.1. Construction of Minimum Bounding Box.

**Fig 3:**
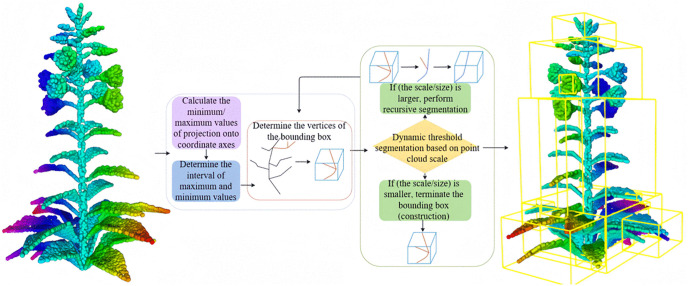
Schematic diagram of axis-aligned minimum bounding box.

An axis-aligned bounding box (AABB) is a cuboid boundary aligned with the global coordinate axes [51]. In 2D space, it is defined by the minimum and maximum x and y coordinates of the input data; in 3D space, it is determined by the minimum and maximum x, y, and z coordinates. We construct an AABB by traversing all point coordinates to identify their minima and maxima, which define the bounding box boundaries. These coordinates form a regular cuboidal space that encloses all discrete points, simplifying complex geometric operations into standardized calculations for regular cuboids. When we use the geometric center of the AABB as the starting point for path planning, it forms the fundamental parameters of the graph structure together with edge weights weighted by Euclidean distance. The principle is shown in Fig 3.

The Euclidean distance-weighted graph structure, constructed using the AABB geometric center, has non-negative edge weights. This property naturally satisfies the core constraint conditions of Dijkstra’s algorithm [52]. We therefore use Dijkstra’s algorithm to solve the 3D shortest distance problem with the AABB geometric center as the source point, achieving optimal distance planning in complex spatial scenarios.

(1) Determination of Bounding Box Geometric Center Starting Point and Euclidean Distance-Weighted Edges

1) We calculate the minimum and maximum values of the point cloud projection on each coordinate axis, as shown in Eq. (13):


$$\begin{array}{c} \left\{ \begin{array}{c} x_{min}=min\left\{x_{i}\right\},\;x_{max}=max\left\{x_{i}\right\}\\ y_{min}=min\left\{y_{i}\right\},\;y_{max}=max\left\{y_{i}\right\}\\ z_{min}=min\left\{z_{i}\right\},\;z_{max}=max\left\{z_{i}\right\} \end{array}\right. \end{array}$$ (13)

The vertices of the bounding box are defined as:


$$\begin{array}{c} \text{AABB}=\{\left(x_{min},y_{min},z_{min}\right),\left(x_{max},y_{max},z_{max}\right)\} \end{array}$$ (14)

2) We iterate through each point to update the bounding box boundaries, following Eq. (15) to Eq. (17):


$$\begin{array}{c} \overset{\left(k\right)}{\underset{min}{x}}=min\left(\overset{\left(k-\text{1}\right)}{\underset{min}{x}},x_{k}\cdot w_{k}\right),\;\overset{\left(k\right)}{\underset{max}{x}}=max(\overset{\left(k-\text{1}\right)}{\underset{max}{x}},x_{k}\cdot w_{k}) \end{array}$$ (15)

$$\begin{array}{c} \overset{\left(k\right)}{\underset{min}{y}}=min\left(\overset{\left(k-\text{1}\right)}{\underset{min}{y}},y_{k}\cdot w_{k}\right),\;\overset{\left(k\right)}{\underset{max}{y}}=max(\overset{\left(k-\text{1}\right)}{\underset{max}{y}},y_{k}\cdot w_{k}) \end{array}$$ (16)

$$\begin{array}{c} \overset{\left(k\right)}{\underset{min}{z}}=min\left(\overset{\left(k-\text{1}\right)}{\underset{min}{z}},z_{k}\cdot w_{k}\right),\;\overset{\left(k\right)}{\underset{max}{z}}=max(\overset{\left(k-\text{1}\right)}{\underset{max}{z}},z_{k}\cdot w_{k}) \end{array}$$ (17)

In these equations, the superscript k denotes the spatial extreme coordinate at the k−th iteration, and $w_{k}$ is the geometric importance weight coefficient of the k−th point. After full traversal, we determine the diagonal vertices from $\left({min}_{x},\;{min}_{y},{min}_{z}\right)$ and $\left({max}_{x},\;{max}_{y},{max}_{z}\right)$, to obtain the boundary of the minimum bounding box.

3) For large-scale point clouds, we recursively divide the minimum bounding box into sub-bounding boxes. For each sub-bounding box ${AABB}_{i}$, we introduce a weight factor $w_{P}$ to screen and retain points based on integrated distance-weighted criteria:


$$\begin{array}{c} p_{\text{keep}}=\text{arg}\underset{p\in {\text{AABB}}_{i}}{min}(w_{P}\cdot ||p-\text{center}\left({\text{AABB}}_{i}\right)\left|\right|) \end{array}$$ (18)

Here, $p_{keep}$ denotes the points retained after weight screening in ${AABB}_{i}$, $w_{P}$ is the weight factor of point p, and $center\left({AABB}_{i}\right)$ is the center point of the sub-bounding box.

4) During the recursive subdivision of the minimum bounding box, we set the stopping condition based on the complexity of the local weight distribution within the sub-bounding boxes. This dynamically adjusts the subdivision granularity and optimizes the allocation of computational resources. Before calculating the weight entropy of the sub-bounding boxes, we normalize the weight factor $w_{P}$ to ensure the rationality of the entropy calculation. First, for all points ${q\in AABB}_{i}$ within the sub-bounding box, we compute the normalized weights as follows:


$$\begin{array}{c} \hat{w_{p}}=\frac{w_{p}}{\sum_{{q\in AABB}_{i}}w_{q}},p\in {AABB}_{i} \end{array}$$ (19)

In Eq. (19),$\;w_{p}\;$denotes the weight factor of a specific point p within the bounding box, $w_{q}$represents the weight factor of any arbitrary point q in the bounding box. $\;\hat{w_{p}}$ stands for the normalized weight, $\sum_{{q\in AABB}_{i}}w_{q}$ is the sum of all weights within the bounding box and ${AABB}_{i}$ refers to the i−th sub-bounding box.

(2) Calculation of Sub-Bounding Box Weight Entropy $H({AABB}_{i})$

We calculate the weight entropy $H({AABB}_{i})$ of the sub-bounding box using Eq. (20):


$$\begin{array}{c} H({AABB}_{i})=-\sum_{p\in {AABB}_{i}}\hat{w_{p}}ln\hat{w_{p}} \end{array}$$ (20)

We compare the weight entropy value $H({AABB}_{i})$ with a predefined threshold ${\;H}_{\text{th}}$. A lower entropy value indicates a more uniform weight distribution, meaning further subdivision provides little additional value. An entropy value above the threshold indicates a complex weight distribution, requiring continued subdivision to distinguish points of varying importance, as expressed in Eq. (21):


$$\begin{array}{c} H({AABB}_{i})<H_{\text{th}} \end{array}$$ (21)

The subdivision process terminates when the weight entropy value $H({AABB}_{i})$ of the sub-bounding box falls below the predefined threshold $H_{\text{th}}$.

(3) Implementation of Dynamic Threshold Subdivision

The dynamic threshold $T_{split}$ is a core parameter for adaptively adjusting the bounding box subdivision granularity, as shown in Eq. (22). Its value is derived from the local curvature entropy model [53], and is adaptively generated by quantifying the confusion degree of weight distribution and point cloud density heterogeneity:


$$\begin{array}{c} T_{split}=\alpha \cdot \overset{―}{H}+\beta \cdot \sigma_{H} \end{array}$$ (22)

Where $\overset{―}{H}$ is the average entropy value of the current-level bounding box, $\sigma_{H}$ is the entropy standard deviation, and α and β are empirical coefficients. Coefficients α and β are optimally determined through grid search experiments conducted on three types of ground object datasets (buildings, vegetation, and roads). The search ranges and step sizes for α and β are specified, and a weighted composite score of feature retention rate and memory compression ratio is adopted as the evaluation metric to finalize the optimal parameters.

If $H({AABB}_{i})>T_{split}$, we continue to recursively subdivide the bounding box; otherwise, we stop subdivision. If the weight difference in the sub-bounding box exceeds the threshold, we trigger non-uniform subdivision, and subdivide high-weight areas first.

#### 2.2.2. Skeleton extraction using MAT Algorithm.

In point cloud processing, the medial axis is defined as the set of centers of maximum inscribed spheres [54]. We extract it through the following steps:

(1) Distance Field Calculation

For each point $p_{i}$, we calculate the distance from this point to the nearest surface point set ∂P:


$$\begin{array}{c} d\left(p_{i}\right)=\underset{p_{j}\in \partial P}{min}|p_{i}-p_{j}| \end{array}$$ (23)

(2) Medial Axis Screening

If $d\left(p_{i}\right)$ is a local maximum in the local neighborhood, we mark it as a medial axis point.

(3) Curvature-Weighted Sampling

We connect the medial axis points to form a simplified skeleton, and assign sampling weights along the skeleton according to curvature:


$$\begin{array}{c} w\left(p_{i}\right)=\frac{\text{1}}{\text{1}+\left|\kappa \left(p_{i}\right)\right|},\;\text{Sampling probability }\propto w\left(p_{i}\right) \end{array}$$ (24)

In Eq. (24), $\kappa \left(p_{i}\right)$ is the curvature at point $p_{i}$, and $\propto w\left(p_{i}\right)$ indicates that the sampling probability is proportional to $w\left(p_{i}\right)$. We search for local maxima in the obtained distance field; these points are the medial axis points of the point cloud.

(4) Medial Axis Topology Construction

**Fig 4:**
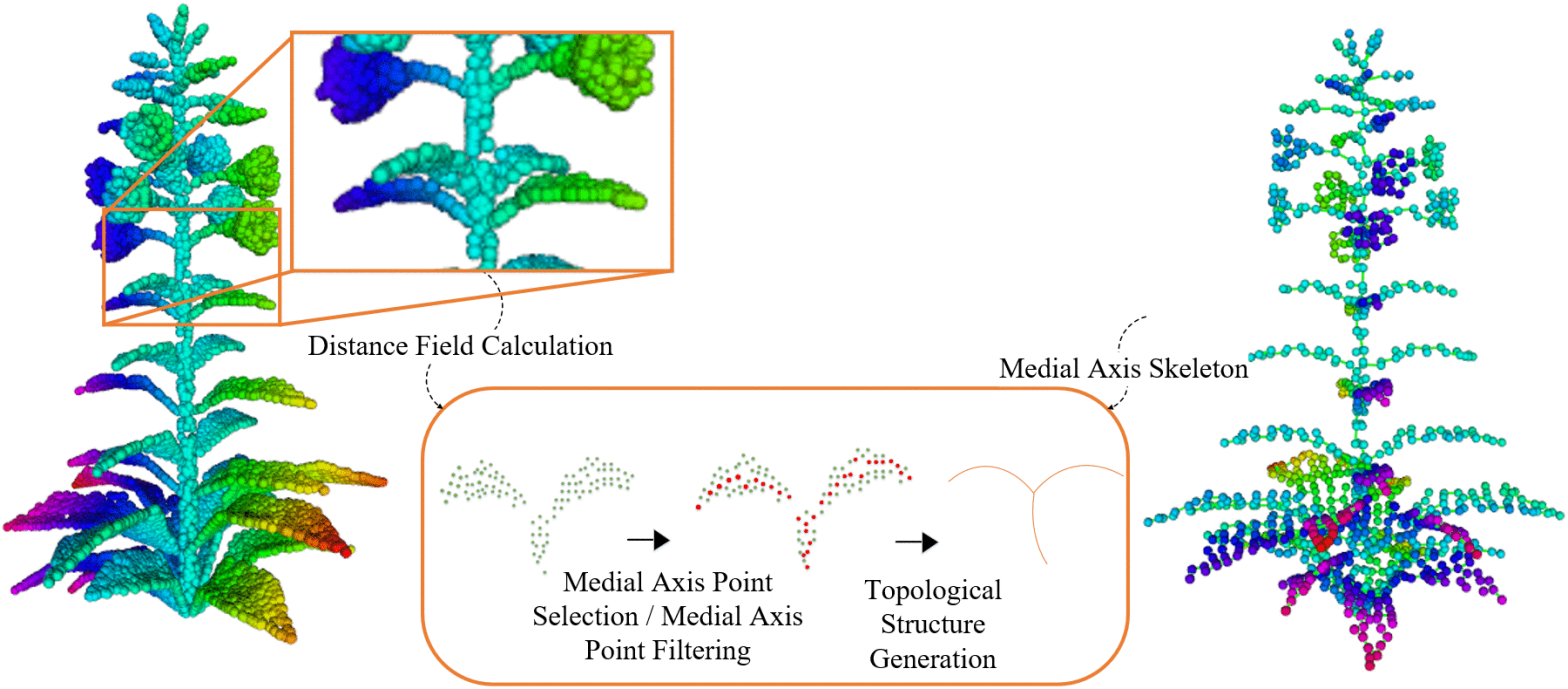
Flowchart of ground objects skeleton extraction.

We generate the topological structure based on the screening results of the medial axis candidate points. First, we use a neighborhood traversal strategy to compare the distance field value of each sampling point with its neighborhood nodes. We define the point set satisfying the local maximum condition as medial axis candidate points. Then, we establish vertex connection relationships when the distance between candidate points meets the threshold condition and the cosine value of the normal angle is greater than the set threshold. This generates a geometrically robust medial axis curve network and topologically shape-preserving medial axis surface structure, as shown in Fig 4.

#### 2.2.3. Medial axis smoothing.

The medial axis derived from the extraction process may contain spurious noise and burrs, which necessitates further smoothing processing. In this study, we first perform initial smoothing of the medial axis point cloud using the moving average method [55]. We update node coordinates based on the arithmetic mean of sample points in the neighborhood window to suppress high-frequency noise. We then apply Gaussian filtering for secondary refinement [56], using a spatial distance-weighted strategy to enhance the weight contribution of adjacent nodes and eliminate interference from discrete outliers. Finally, we implement topological simplification via the Douglas-Peucker algorithm [57]. We iteratively calculate the maximum deviation between sampling points and the reference line to dynamically retain key feature points and eliminate redundant nodes, ultimately obtaining a geometric expression of the medial axis with a simplified structure.


$$\begin{array}{c} \sigma =\frac{W_{{edge}_{parent}}}{W_{{edge}_{v_{\text{0}}}}}\times R_{v_{\text{0}}} \end{array}$$ (25)

In Eq. (25), σ represents the standard deviation of the adaptive Gaussian filter kernel function, $W_{{edge}_{parent}}$ is the weight of the current edge, $W_{{edge}_{v_{\text{0}}}}$ is the weight of the edge where the root node of the skeleton is located, and $R_{v_{\text{0}}}$ is the trunk radius.

### 2.3. Normal Estimation and Correction

Sliding window filtering effectively denoises and smooths point cloud data, but may compromise the preservation of sharp geometric features. For this reason, we perform targeted screening of sharp feature points and their adjacent points to support targeted normal correction.

#### 2.3.1. Sharp Feature Point Identification.

First, we calculate geometric feature statistics in the local neighborhood: normal vector angle, elevation standard deviation, and curvature distribution. We quantify the local curvature and terrain undulation intensity, and construct a multi-dimensional feature space to describe local geometric characteristics. We calculate the terrain undulation intensity using Eq. (26) [58]:


$$\begin{array}{c} \sigma_{z}=\sqrt{\frac{\text{1}}{N}\overset{N}{\underset{K=\text{1}}{\Sigma }}{\left(z_{k}-\overset{―}{z}\right)}^{\text{2}}} \end{array}$$ (26)

In Eq. (26), $\sigma_{z}$ denotes the terrain undulation intensity, N is the number of valid points involved in the elevation standard deviation calculation in the current local neighborhood, $z_{k}$ is the elevation value of the k−th point in the local neighborhood, and $\overset{―}{z}$ is the average elevation of the neighborhood.

Combining point cloud distribution characteristics and empirical data, we establish an adaptive threshold adjustment mechanism: we use a lenient threshold for regions with large terrain undulation to enhance feature inclusiveness, and a strict threshold for flat regions to reduce the false detection rate. On this basis, the k-neighborhood point set is extracted based on spatial proximity, which are the screened sharp feature points. The lenient threshold is calculated using Eq. (27), and the strict threshold is computed via Eq. (28):


$$\begin{array}{c} T_{loose}=\sqrt{\mu_{R}-\alpha \cdot \sigma_{R}} \end{array}$$ (27)

where $\mu_{R}$ denotes the mean value of terrain undulation intensity of the current point cloud, $\sigma_{R}$ represents the standard deviation of terrain undulation intensity, and α is an adjustment coefficient with a value range of [1.0, 1.5].


$$\begin{array}{c} T_{loose}=\sqrt{\mu_{R}-\beta \cdot \sigma_{R}} \end{array}$$ (28)

where $\mu_{R}$ denotes the mean value of terrain undulation intensity of the current point cloud, $\sigma_{R}$ represents the standard deviation of terrain undulation intensity, and β is an adjustment coefficient with a value range of [0.5, 1.0).

#### 2.3.2. Normal correction.

We first construct a feature propagation path based on dihedral angle geometric constraints [59]. This path is iteratively extended along the edge vector direction of the candidate point’s neighborhood, with a step size equal to the neighborhood radius. We simultaneously verify the consistency of normal directions between consecutive extended points. Propagation is terminated when the normal vector difference between adjacent extended points falls below the predefined tolerance, indicating that the plane stable region has been reached.

We then correct the normal direction of path nodes via reverse propagation, to achieve a continuous and smooth transition of the normal field at feature edges. The formula for normal direction consistency detection is defined as:


$$\begin{array}{c} |n_{t}-n_{t-\text{1}}|\leq \theta_{threshold} \end{array}$$ (29)

In Eq. (29), $n_{t}$ and $n_{t-\text{1}}$ denote the normals of adjacent extended points in the propagation path, and $\theta_{threshold}$ is the normal difference tolerance threshold.

### 2.4 Algorithm complexity analysis

To systematically evaluate the engineering practicality of the proposed method, we conducted a quantitative analysis of the time and memory complexity of each module, considering three core variables: the point cloud scale N, the number of neighborhood search points k (a constant), and the number of recursive partitioning levels h of the AABB bounding boxes.

#### 2.4.1 Time complexity.

Adaptive sliding window polynomial fitting filter: Each point only requires a single window traversal and fixed-order polynomial fitting, with a time complexity of O(N).

Entropy-driven adaptive AABB recursive partitioning: Each point only participates in one bounding box assignment and entropy calculation, with a time complexity of O(N).

Curvature-weighted enhanced MAT skeleton extraction: The dominant computational cost comes from KD-Tree accelerated neighborhood search and distance field calculation, with a time complexity of O(NlogN).

Normal estimation and correction: The computational cost comes from local PCA analysis and neighborhood traversal, with a time complexity of O(NlogN).

Overall, the dominant term of the algorithm’s time complexity is O(NlogN).

#### 2.4.2. Memory complexity.

Adaptive sliding window filter: Only occupies a fixed-size window cache, with a space complexity of O(1).

Entropy-driven AABB partitioning: The number of partitioning levels h follows h=O(logN), and the storage of sub-bounding boxes has a worst-case space complexity of O(N). In practical urban point cloud processing, the number of partitioning levels is limited, and the actual space complexity is O(logN), which is a low-order term with negligible impact on overall memory consumption.

Skeleton extraction and normal estimation modules: Only need to store linear-scale attribute data (coordinates, normals, curvature), with a space complexity of O(N) for both.

Overall, the algorithm has an overall linear memory complexity of O(N) with no redundant memory overhead, belonging to the high memory efficiency class in the field of point cloud processing.

These complexity characteristics demonstrate that the proposed method can simultaneously guarantee computational accuracy, processing efficiency, and memory resource utilization efficiency when processing large-scale point cloud data, and thus has strong potential for practical engineering applications.

## 3. Experiments and discussion

The experiment was conducted at the Huifeng Campus of Chuzhou University, Chuzhou, Anhui Province, which covers a total area of 294,918 m^2^. Its geographic coordinates range from 118°18′43″ E to 118°18′44″ E, and from 32°16′39″ N to 32°16′40″ N. The point cloud dataset used in the experiment contains 78,675,354 points, with a point cloud density of 114,132 points/m^3^. Based on the classification standard of the International Society for Photogrammetry and Remote Sensing (ISPRS), we divide the point cloud in this area into three datasets of ground object elements (details in Table 1). We implemented the core experimental programs in Visual Studio 2022, PCL 1.14.1, and C++17 development environments. The hardware platform is configured with an Intel Core i7-10700K CPU (3.8 GHz), 32 GB of RAM, and an NVIDIA RTX 3080 GPU, running Microsoft Windows 11.

**Table 1:** Detailed information of experimental datasets.

Typical structure	Feature description		Density range (points/m²)
1)Building	Characterized by regular geometric edges, planar orthogonality, and spatial symmetry. They exhibit a rigid connectivity structure with strongly dominant principal directions.		14500 ~ 89000
2)Vegetation	Defined by a fractal, tree-like structure with multi-scale details (from trunks to branches to leaves). It consists of hierarchical, connected branches with rapidly varying local curvature.		2700 ~ 12600
3)Road	Feature a band-like, elongated structure with smooth, gradual transitions in cross-section. Characterized by a banded, elongated structure with smooth, gradual cross-sectional transitions. Connectivity is primarily linear, with overpasses forming supporting structures and layered regions exhibiting trapezoidal morphological characteristics.		1800 ~ 11200

### 3.1. Point cloud filtering analysis

**Fig 5:**
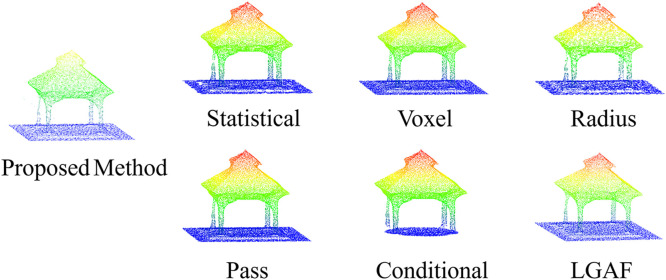
Comparison of filtering effects of different algorithms (Dataset 1).

**Fig 6:**
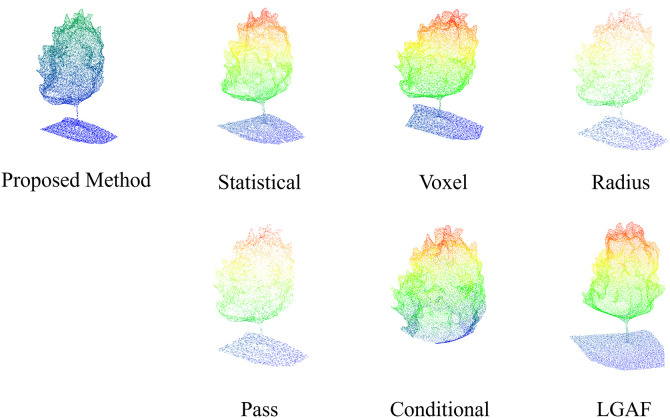
Comparison of filtering effects of different algorithms (Dataset 2).

**Fig 7:**
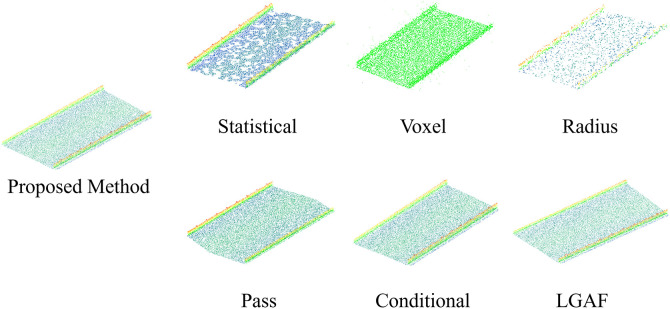
Comparison of filtering effects of different algorithms (Dataset 3).

To quantitatively evaluate the performance of the proposed adaptive sliding window polynomial fitting filter, we designed a series of comparative experiments. Five conventional filtering methods were selected as comparative benchmarks: Statistical Outlier Filtering (SOR), Voxel Grid Filtering, Radius Outlier Filtering, Pass-through Filtering, and Conditional Filtering. Local Gradient-Aware Filtering (LGAF) [60] was also included in the comparison. Quantitative results are summarized in Tables 2–4, and the visual filtering effects are shown in Fig 5, Fig 6, Fig 7.

**Table 2:** Comparison of filtering performance of different algorithms (Dataset 1).

Indicator\Algorithm	Statistical	Voxel	Radius	Pass	Conditional	LGAF	Proposed Method
Noise removal rate (%)	88.7 ± 1.2	65.3 ± 2.5	88.2 ± 1.5	66.2 ± 2.3	72.4 ± 2.1	88.9 ± 1.1	89.2 ± 1.0
Effective point retention rate (%)	90.8 ± 1.1	95.3 ± 0.7	83.7 ± 1.8	91.0 ± 1.0	80.3 ± 2.0	91.1 ± 0.4	91.2 ± 1.2
Edge retention rate (%)	76.5 ± 2.3	75.2 ± 2.2	80.1 ± 1.7	79.8 ± 1.6	60.3 ± 3.1	84.2 ± 1.2	82.5 ± 2.0
Processing time (ms)	120.0 ± 5.0	85.0 ± 10.0	145.0 ± 6.0	180.0 ± 7.0	50.0 ± 2.0	105.0 ± 4.0	95.0 ± 6.0

**Table 3:** Comparison of filtering performance of different algorithms (Dataset 2).

Indicator\Algorithm	Statistical	Voxel	Radius	Pass	Conditional	LGAF	Proposed Method
Noise removal rate (%)	75.3 ± 2.0	67.1 ± 2.6	90.7 ± 1.5	72.5 ± 2.3	92.1 ± 1.2	88.9 ± 2.1	88.4 ± 1.6
Effective point retention rate (%)	85.8 ± 1.3	89.4 ± 0.8	42.8 ± 3.2	88.5 ± 1.1	62.6 ± 2.8	90.1 ± 1.5	87.9 ± 1.0
Edge retention rate (%)	79.6 ± 1.7	80.2 ± 1.5	75.4 ± 2.1	81.4 ± 1.4	70.1 ± 2.8	84.5 ± 2.6	81.8 ± 1.3
Processing time (ms)	157.0 ± 6.0	120.0 ± 4.0	49.0 ± 2.0	172.0 ± 5.0	55.0 ± 8.0	94.0 ± 5.0	88.0 ± 6.0

**Table 4:** Comparison of filtering performance of different algorithms (Dataset 3).

Indicator\Algorithm	Statistical	Voxel	Radius	Pass	Conditional	LGAF	Proposed Method
Noise removal rate (%)	83.4 ± 1.6	75.9 ± 2.2	89.7 ± 1.4	65.8 ± 2.5	84.6 ± 1.7	86.2 ± 1.3	85.7 ± 1.5
Effective point retention rate (%)	87.4 ± 2.3	86.5 ± 1.4	64.0 ± 2.8	88.4 ± 1.6	92.5 ± 0.9	92.8 ± 1.4	88.9 ± 1.0
Edge retention rate (%)	75.4 ± 2.0	79.4 ± 1.8	68.9 ± 2.6	71.5 ± 2.3	85.4 ± 1.5	90.4 ± 2.1	85.6 ± 1.4
Processing time (ms)	80.0 ± 9.0	43.0 ± 5.0	28.0 ± 6.0	74.0 ± 11.0	36.0 ± 5.0	52.0 ± 7.0	42.0 ± 4.0

Filtering results for building point clouds in Table 2 demonstrate that the proposed method achieves a noise removal rate of 89.2%, outperforming all other comparative approaches. For inlier retention ratio, Voxel Grid Filtering delivers the highest performance at 95.3%, followed by our proposed method at 91.2%. In terms of edge preservation, LGAF achieves the optimal performance at 84.2%, with our method closely following at 82.5% — a value significantly higher than all other comparative algorithms. Conditional Filtering yields the fastest processing speed. When evaluated across all metrics comprehensively, the overall performance of the proposed method exceeds that of all other tested approaches.

Analysis of the filtering results for vegetation point cloud data (Table 3) reveals that Conditional filtering achieves the highest noise removal rate, at 92.1%. The LGAF algorithm delivers the highest inlier retention rate at 90.1%, followed by Voxel filtering at 89.4% and the proposed method at 87.9%. With regard to edge preservation, the LGAF algorithm performs optimally at 84.5%, and the proposed method ranks second at 81.8%.

From the filtering results of road point cloud data (Table 4), we observe that Radius filtering achieves the highest noise removal rate, at 89.7%. For inlier retention, the LGAF algorithm delivers the best performance at 92.8%, followed by Conditional filtering at 92.5%, with the proposed method ranking third at 88.9%. In terms of edge preservation, the LGAF algorithm performs optimally at 90.4%, and the proposed method ranks second at 85.6%, outperforming all other comparative algorithms.

To ensure the statistical reliability of the results, each comparative experiment was independently repeated 5 times under identical initial conditions. We performed analysis of variance (ANOVA) followed by Tukey’s Honestly Significant Difference (HSD) post-hoc test for significance testing, to evaluate the statistical differences between the tested methods. A p-value of less than 0.05 was considered statistically significant.

### 3.2. Skeleton extraction analysis

**Fig 8:**
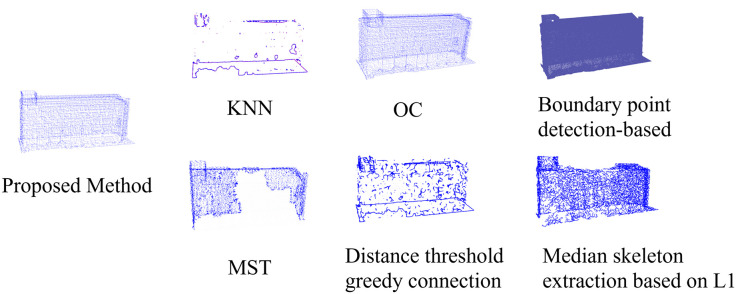
Medial axis skeleton extraction results (Dataset 1).

**Fig 9:**
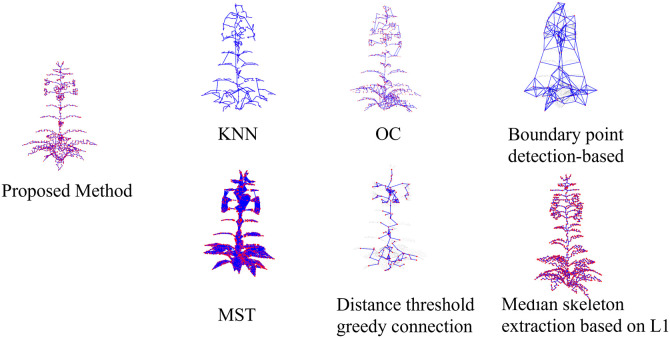
Medial axis skeleton extraction results (Dataset 2).

**Fig 10:**
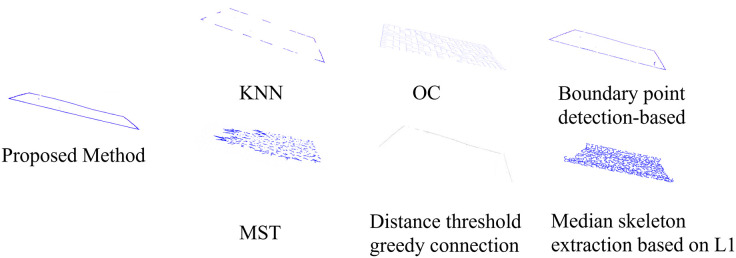
Medial axis skeleton extraction results (Dataset 3).

To systematically evaluate the proposed skeleton extraction algorithm integrating minimum bounding boxes and medial axis transform, we selected six comparative methods: the KNN algorithm, MST algorithm, orientation consistency (OC) algorithm, boundary point detection-based algorithm, distance threshold greedy connection algorithm, and L1-based median skeleton extraction algorithm. We conducted quantitative comparisons across three core dimensions: topological accuracy, geometric fidelity, and computational efficiency. We introduced three key metrics for the topological accuracy dimension. The first is topology integrity, which measures the matching consistency between the extracted skeleton and the true topological structure of the original point cloud. The second is branch accuracy, which reflects the algorithm’s ability to accurately identify complex branch morphologies such as vegetation and building accessory structures. The third is connectivity score, which evaluates the connection continuity between skeleton nodes and the integrity of the overall topological structure. The medial axis skeleton extraction results of all algorithms are illustrated in Fig 8, Fig 9, Fig 10.

The formula for topology integrity is given by:


$$\begin{array}{c} TopoIntegrity=\text{1}-\frac{\left|\tau_{skel}⊖\tau_{cloud}\right|}{\left|\tau_{cloud}\right|} \end{array}$$ (30)

In this equation, TopoIntegrity denotes topology integrity. $\tau_{cloud}$ represents the set of topological features extracted from the original point cloud via a standardized method, including the number of branch points, endpoints, and independent loops. $\tau_{skel}$ is the corresponding set of topological features derived from the extracted skeleton. The operator ⊖ denotes the symmetric difference, defined as the count of elements that belong to exactly one of the two sets rather than their intersection. This metric quantifies the discrepancy between the two sets of topological features. $\tau_{cloud}$, the cardinality of the topological feature set of the original point cloud, acts as the normalization factor. This formula produces a score in the range of [0, 1]. A value closer to 1 indicates that the skeleton retains topological features consistent with those of the original point cloud. The symmetric difference increases and the score decreases if the skeleton contains false additional branches or is missing genuine branches present in the original point cloud.

The formula for branch accuracy is given by:


$$\begin{array}{c} BranchAccuracy=\text{2}\cdot \frac{{Precision}_{branch}\cdot {Recall}_{branch}}{{Precision}_{branch}+{Recall}_{branch}} \end{array}$$ (31)

In this equation, BranchAccuracy denotes branch accuracy. ${Precision}_{branch}$ is calculated as the number of correctly identified branches divided by the total number of branches in the extracted skeleton. ${Recall}_{branch}$ is calculated as the number of correctly identified branches divided by the total number of branches in the original point cloud. A correctly identified branch is defined as a branch whose nodes match the corresponding genuine branch nodes in the original point cloud in both spatial position and adjacency relationships.

The formula for the connectivity score is given by:


$$\begin{array}{c} ConnectivityScore=\frac{{CC}_{matched}}{{CC}_{total}}\cdot \left(\text{1}-\frac{NumGaps}{{NumEdges}_{ideal}}\right) \end{array}$$ (32)

In this equation, ConnectivityScore denotes the connectivity score. ${CC}_{matched}$ refers to the number of connected components in the skeleton graph that match the corresponding components in the original point cloud. ${CC}_{total}$ is the total number of connected components in the original point cloud. NumGaps represents the number of gaps in the skeleton, where structures that should be physically contiguous are in fact fractured. ${NumEdges}_{ideal}$ is the ideal number of edges for a fully connected topology.

For three typical ground objects– the regular edges of building facades, fractal structures of vegetation, and strip-shaped features of roads – we established a multi-scale evaluation system. Specifically, we adopted high-density control point sampling to achieve registration accuracy within 0.5 mm. We also designed point cloud density gradients (50–500 points/m²) and Gaussian noise perturbations (σ = 0.05–0.2 m) to verify the algorithm’s stability.

In terms of geometric and topological accuracy (Table 5), the proposed method achieves a topology integrity of 0.93 and a branch accuracy of 0.95 for building ground objects. These values correspond to a 9.4% and 8.0% improvement, respectively, compared to the orientation consistency (OC) algorithm, with a node fracture ratio as low as 2.1%–3.5%. For the fine-branch structures of vegetation and the banded structures of roads, our method attains a direction consistency score of 0.96, a 47.7% increase over the MST algorithm. This result demonstrates its ability to accurately characterize the topological structures of diverse ground object types. The L1-based median skeleton extraction algorithm exhibits favorable performance in directional consistency (0.84) and resolution robustness (0.88). Even so, a significant gap remains between this algorithm and the proposed method in terms of branch accuracy (0.74) and topology integrity (0.82). This finding suggests that its ability to preserve topological fidelity requires further improvement when processing complex branch structures.

**Table 5:** Comparison of geometric topology evaluation indicators for skeleton extraction.

Indicator\Algorithm	KNN	MST	OC	Boundary point detection-based	Distance threshold greedy connection	Median skeleton extraction based on L1	Proposed Method
Topology integrity	0.68	0.72	0.85	0.85	0.78	0.82	0.93
Branch accuracy	0.63	0.69	0.88	0.55	0.72	0.74	0.95
Directional Consistency	0.60	0.65	0.92	0.52	0.68	0.84	0.96
Coverage	0.72	0.75	0.80	0.92	0.62	0.78	0.94
Connectivity score	0.70	0.92	0.83	0.60	0.75	0.79	0.95

Table 6 presents the robustness evaluation results. When Gaussian noise with σ = 0.1 m is introduced, the proposed algorithm maintains a noise robustness of 0.92, which is 27.8% higher than that of the MST algorithm. It also achieves a parameter sensitivity score of 0.85, a 142.9% improvement compared to the distance threshold greedy algorithm. These findings indicate the algorithm’s stable performance under complex noise interference and parameter fluctuations. The L1-based median skeleton extraction algorithm outperforms both the MST and KNN algorithms with respect to noise robustness (0.84) and resolution robustness (0.88). Conversely, a measurable gap remains between this algorithm and the proposed method in terms of parameter sensitivity (0.80), a result that reflects its inherent limitations in adaptive parameter tuning capability.

**Table 6:** Performance evaluation of medial axis skeleton extraction.

Indicator\Algorithm	KNN	MST	OC	Boundary point detection-based	Distance threshold greedy connection	Median skeleton extraction based on L1	Proposed Method
Run time (s/1000points)	0.05	0.12	0.25	0.08	0.15	0.11	0.10
Noise robustness(σ = 0.1m)	0.65	0.72	0.82	0.58	0.75	0.84	0.92
Resolution robustness	0.60	0.68	0.78	0.50	0.72	0.88	0.89
parameter sensitivity	0.55	0.60	0.45	0.40	0.35	0.80	0.85

The comparative results for computational efficiency are shown in Table 7. Leveraging the hierarchical axis-aligned bounding box (AABB) adaptive recursive partitioning strategy, the proposed algorithm processes million-point-scale data in only 5.2 seconds—29.7% faster than the KNN algorithm—with a real-time performance of 18.7 fps and a memory footprint of merely 110 MB. While the KNN algorithm exhibits the fastest speed for small-scale data processing (0.05 seconds per thousand points), it suffers from poor scalability. In contrast, our method effectively balances processing efficiency and data scale adaptability through multi-resolution optimization and adaptive sampling strategies. The L1-based median skeleton extraction algorithm exhibits favorable performance in terms of extensibility index (0.90) and adaptability to complex structures (0.75). Despite this, a significant discrepancy persists between this algorithm and the proposed method with regard to processing efficiency (12.5 s per million points) and real-time performance (9.8 fps). It also incurs a higher memory footprint (160 MB). These findings indicate that there remains scope for optimization in the computational resource consumption of this algorithm.

**Table 7:** Algorithm complexity and scalability evaluation.

Indicator\Algorithm	KNN	MST	OC	Boundary point detection-based	Distance threshold greedy connection	Median skeleton extraction based on L1	Proposed Method
Million Point Processing Time (s)	7.40	125.60	18.20	10.80	13.00	12.50	5.20
Extensibility index	0.75	0.35	0.68	0.72	0.70	0.90	0.92
Real-time performance (fps)	12.50	3.20	8.30	10.20	9.10	9.80	18.70
Memory occupancy (MB)	80.00	120.00	180.00	95.00	130.00	160.00	110.00
Adaptability of complex structure	0.62	0.70	0.84	0.53	0.76	0.75	0.93

Overall, the proposed method outperforms other algorithms in core geometric and topological metrics such as topology integrity, branch correctness, and direction consistency. Although the direction consistency algorithm performs well in direction preservation (0.92), it shows significant deficiencies in topology integrity (0.85) and connectivity (0.83), failing to meet the topological fidelity requirements for point clouds of complex ground objects. The L1-based median skeleton extraction algorithm delivers moderate performance in terms of resolution robustness (0.88) and parameter sensitivity (0.80). There is, however, a significant gap between this algorithm and the proposed method with respect to branch correctness (0.74) and adaptability to complex structures (0.75). This finding indicates that the algorithm still suffers from insufficient topological fidelity when processing complex branch structures.

To ensure the statistical reliability of the skeleton extraction experimental results, all comparative experiments were independently repeated 5 times under identical initial conditions. One-way analysis of variance (ANOVA) was performed to assess the overall differences between the tested algorithms across key metrics including topology integrity, branch correctness, and directional consistency. Tukey’s Honestly Significant Difference (HSD) post-hoc test was further applied for multiple comparisons to identify significant differences between each pair of algorithms. A p-value of less than 0.05 was considered statistically significant.

### 3.3. Normal estimation analysis

**Fig 11:**
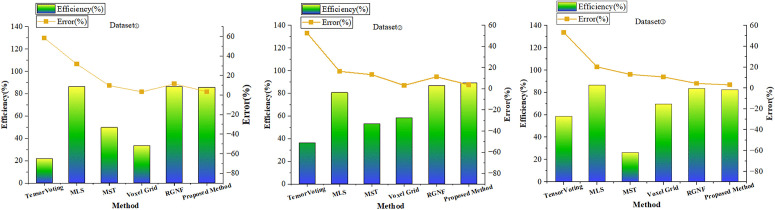
Analysis of normal calculation efficiency and error.

**Fig 12:**
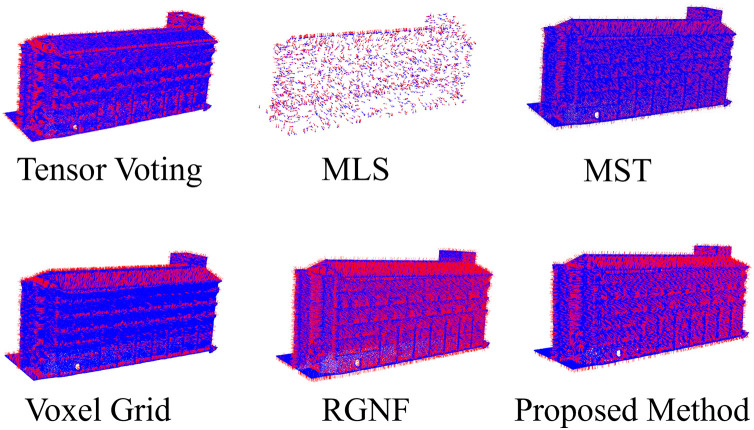
Normal estimation results (Dataset 1).

**Fig 13:**
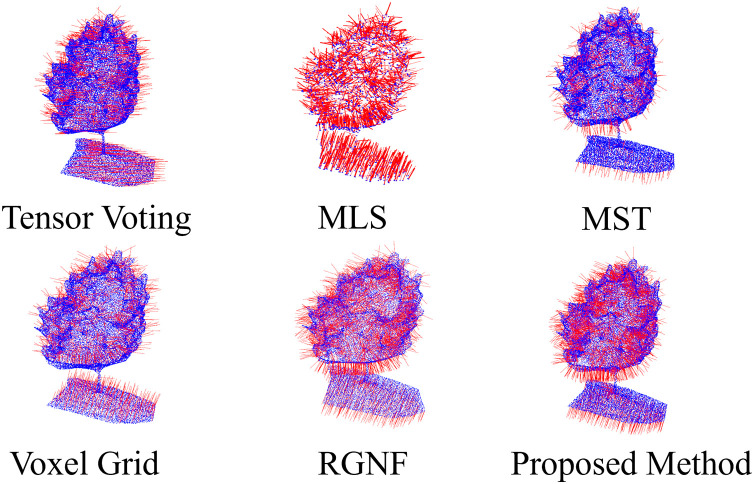
Normal estimation results (Dataset 2).

**Fig 14:**
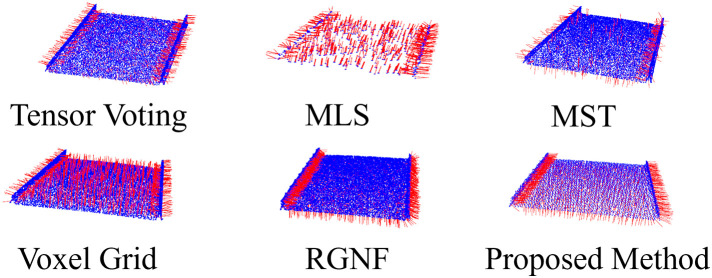
Normal estimation results (Dataset 3).

To evaluate the performance of the proposed normal estimation algorithm, we established a standardized comparative experimental framework. Using the same point cloud dataset, we systematically compared the proposed method with five existing algorithms: Tensor Voting, MST, Voxel Grid, Robust Gaussian Normal Fitting (RGNF), and MLS. The evaluation dimensions include normal vector calculation efficiency (point cloud data processed per unit time), computational accuracy (quantified by a feature-sensitive point set weighted scoring method, where weights are assigned according to the local geometric complexity of the point cloud), and computation time. The experimental results are presented in Tables 8, 9 and 10. We also provide an intuitive analysis of the efficiency and error in normal vector calculations in Fig 11, and visual comparisons of normal estimation results in Fig 12, Fig 13, Fig 14.

**Table 8:** Comparison of normal calculation efficiency (%).

Data\algorithm	Tensor Voting	MLS	MST	Voxel Grid	RGNF	Proposed Method
Dataset1	21.76	86.35	49.69	33.62	86.53	85.62
Dataset2	36.30	80.67	53.25	58.36	86.76	89.14
Dataset3	58.58	86.54	26.35	69.65	83.51	82.36
Average Value	38.88	84.52	40.09	53.87	85.60	85.70

**Table 9:** Comparison of normal calculation error (%).

Data\algorithm	Tensor Voting	MLS	MST	Voxel Grid	RGNF	Proposed Method
Dataset1	58.34	31.53	9.57	3.31	11.36	3.42
Dataset2	52.45	16.35	13.24	3.13	11.24	3.59
Dataset3	53.26	20.23	12.97	10.56	4.35	3.02
Average Value	54.68	22.70	11.92	5.66	8.98	3.34

**Table 10:** Comparison of normal calculation time consumption (s).

Data\ algorithm	Tensor Voting	MLS	MST	Voxel Grid	RGNF	Proposed Method
Dataset1	15.968	8.218	10.695	10.708	10.286	9.859
Dataset2	10.824	7.329	8.487	8.837	8.605	8.258
Dataset3	10.560	7.872	8.744	8.912	9.458	8.298
Average Value	12.451	7.806	9.309	9.485	9.450	8.805

(1) Dataset 1: This dataset primarily covers buildings, with point clouds exhibiting regular geometric edges, planar orthogonality, and spatial symmetry. The structures are rigidly connected with distinct main directions.

The normal estimation results for building point cloud data in Tables 8 and 9 reveal that the RGNF algorithm achieves the optimal efficiency at 86.53%, followed closely by the MLS algorithm (86.35%) and the proposed method (85.62%). Regarding normal estimation error, the Voxel Grid algorithm yields the smallest error at approximately 3.31%, with the proposed method ranking second.

(2) Dataset 2: Dominated by vegetation, the point clouds in this dataset feature fractal tree-like structures with multi-scale details (trunk-branch-leaf). They exhibit hierarchical connected branches and significant local curvature variations.

From the normal estimation results of vegetation point cloud data (Tables 8 and 9), the proposed method demonstrates the best computational efficiency at 89.14%. For normal estimation error, the Voxel Grid algorithm has the smallest error at only 3.13%—the proposed method’s error is merely 0.46% higher, yet its computational efficiency is significantly superior to that of the Voxel Grid algorithm.

(3) Dataset 3: The point clouds here present strip-shaped extended structures with smoothly gradient cross-sections, primarily linear connectivity, load-bearing structures at overhanging positions, and trapezoidal morphological features in layered areas.

As indicated by the normal estimation results of road point cloud data (Tables 8 and 9), the MLS algorithm achieves the highest computational efficiency at 86.54%. In terms of normal estimation error, the proposed method performs optimally with an error of only 3.02%.

### 3.4. 3D reconstruction evaluation

In this experiment, we used CloudCompare software for 3D reconstruction of the downsampled point cloud data from each algorithm. We adopted three key metrics—geometric fidelity, structural integrity, and computational efficiency—to comprehensively evaluate the 3D reconstruction results of the downsampled point clouds.

For geometric fidelity assessment, we selected the average point-to-surface distance (APtSD) and maximum point-to-surface distance (MPtSD) as quantitative indicators. The APtSD, calculated using Eq. (33), represents the mean distance from all points in the point cloud to the surface of the reconstructed model. This metric effectively measures the overall geometric deviation and surface smoothness of the model. The MPtSD is defined as the largest deviation among all sampled points from the reconstructed surface, i.e., $d_{max}=\text{max}(d_{\text{1}},d_{\text{2}},\ldots ,d_{N})$, and reflects the degree of local geometric distortion in the reconstruction results.


$$\begin{array}{c} \overset{―}{d}=\frac{\text{1}}{N}\sum_{i=\text{1}}^{N}d_{i} \end{array}$$ (33)

In the equation, N denotes the total number of sampled points, and $d_{i}$ is the distance from the i−th sample point to the reconstructed surface.

For structural integrity, we used the fracture rate to quantify the loss of structural information during reconstruction, with the calculation formula given in Eq. (34).


$$\begin{array}{c} R_{f}=\frac{M}{K}\times \text{1}\text{0}\text{0}\% \end{array}$$ (34)

In the equation, M is the number of fractured structures in the reconstruction results, and K is the total number of intact structures in the original model.

Regarding computational efficiency, we mainly statistically analyzed the reconstruction time and memory usage of each method. These two indicators were used to comprehensively evaluate differences in computational resource consumption among different methods.

**Fig 15:**
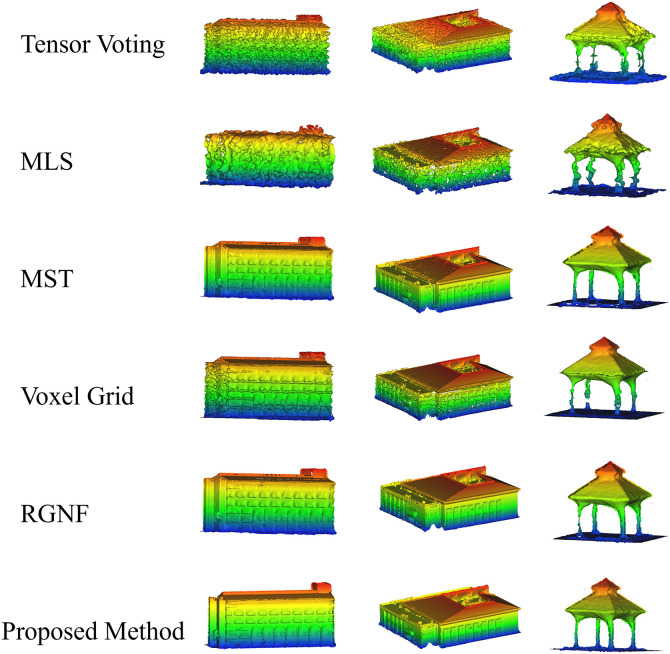
3D reconstruction results of different algorithms (Buildings).

**Fig 16:**
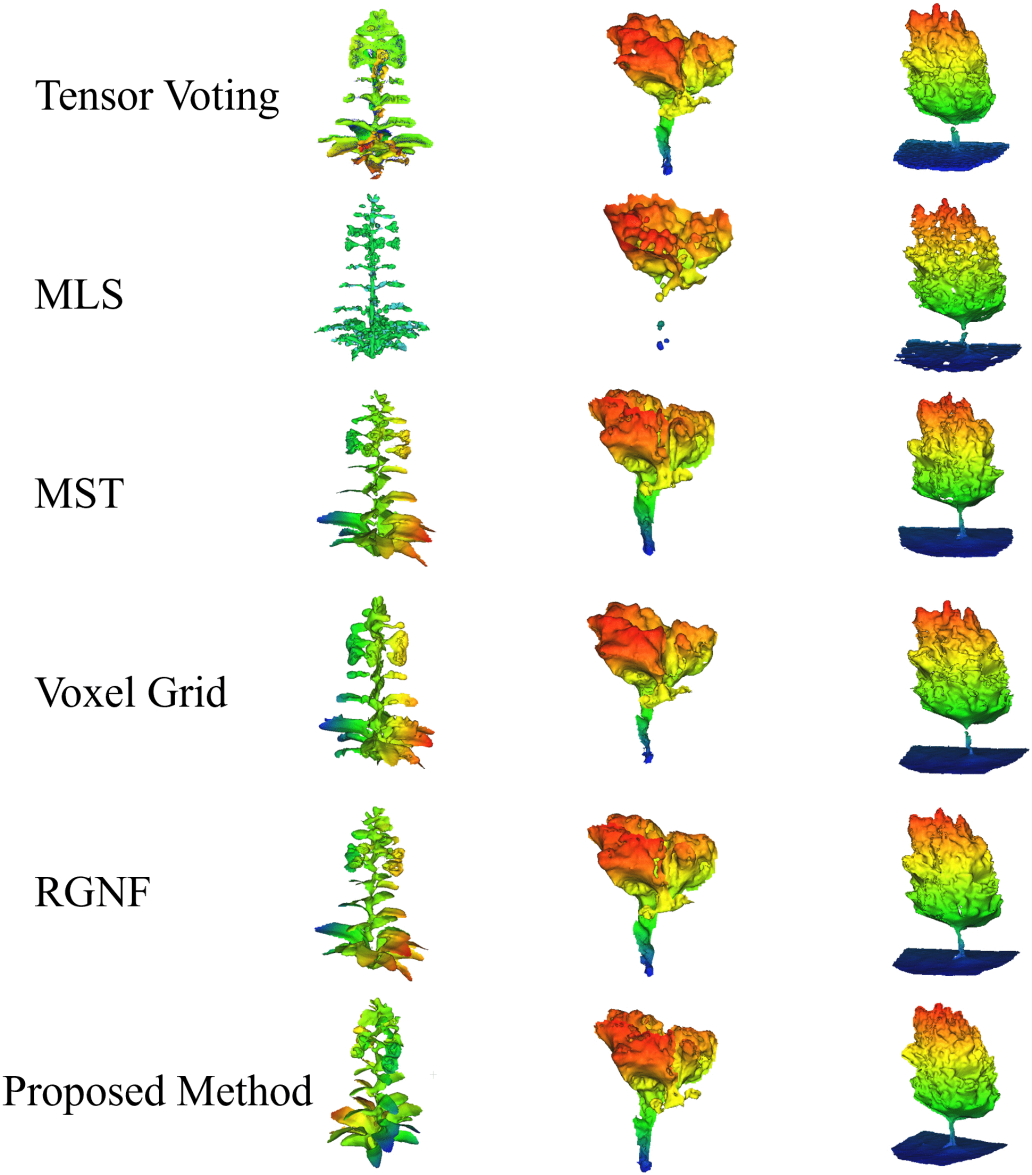
3D reconstruction results of different algorithms (Vegetation).

**Fig 17:**
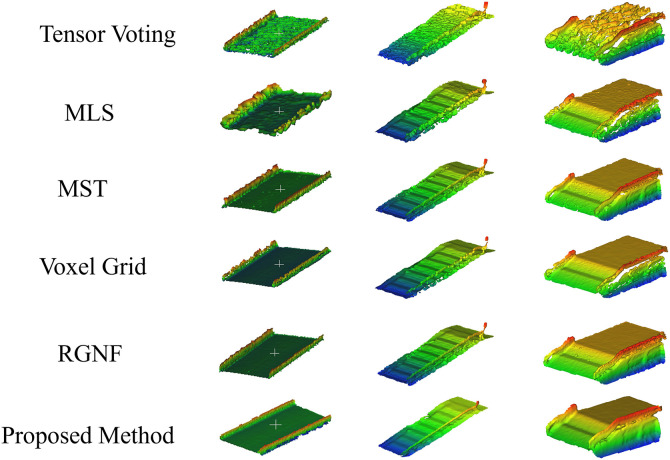
3D reconstruction results of different algorithms (Roads).

**Fig 18:**
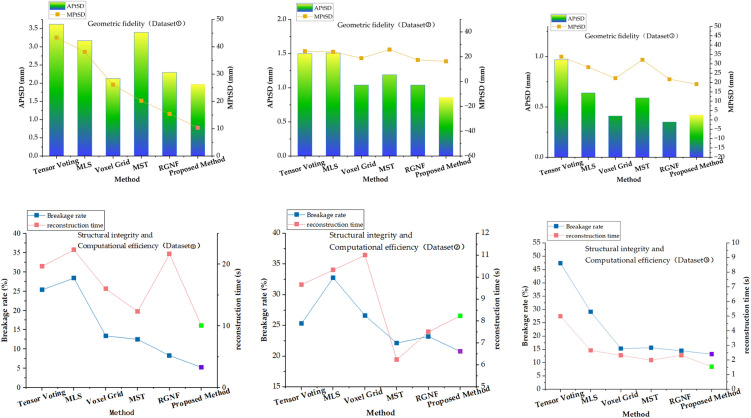
Analysis of 3D reconstruction effects of three types of ground objects.

Tables 11, 13, and 15 list the original point cloud sizes of three selected ground objects in different datasets and the downsampled point cloud sizes from each algorithm, respectively. The 3D reconstruction results are illustrated in Figs 15–17. Fig 18 provides a visual analysis of the three-dimensional reconstruction results for the three types of terrain features.

**Table 11:** Number of point clouds after lightweighting by different algorithms (Buildings).

Data\algorithm	Point Count	Tensor Voting	MLS	MST	Voxel Grid	RGNF	Proposed Method
Buildings1	862358	800000	322073	696265	732351	741574	423868
Buildings2	785124	700000	421525	596545	625111	522488	395412
Buildings3	25486	20000	9347	16542	20301	15661	8967

**Table 12:** Evaluation of 3D reconstruction results (Dataset 1).

Method\indicator	Geometric fidelity(mm)		Structural integrity	Computational efficiency	
	APtSD	MPtSD	Breakage rate (%)	reconstruction time(s)	Memory occupancy (MB)
Tensor Voting	3.62	43.37	25.35	19.66	133.00
MLS	3.17	38.12	28.39	22.33	120.00
MST	2.13	26.09	13.40	16.00	97.33
Voxel Grid	3.40	20.23	12.51	12.33	102.00
RGNF	2.30	15.44	8.30	21.66	92.33
Proposed Method	1.97	10.34	5.24	10.05	102.66

**Table 13:** Number of points in lightweighted point clouds by different algorithms (Vegetation).

Data\algorithm	Point Count	Tensor Voting	MLS	MST	Voxel Grid	RGNF	Proposed Method
Vegetation1	10562	10000	7300	8652	5327	8691	6778
Vegetation2	8052	5900	5408	5620	4242	4799	4330
Vegetation3	43960	43000	17738	37620	38656	41356	30782

The evaluation metrics for 3D reconstruction results of building point cloud data in Table 12 reveal the following: In terms of geometric fidelity, the Tensor Voting algorithm exhibits the largest APtSD at 3.62 mm and the largest MPtSD at 43.37 mm. In stark contrast, the proposed method achieves the smallest values for both metrics, at 1.97 mm and 10.34 mm respectively. Regarding structural integrity, the MLS algorithm has the highest fracture rate at approximately 28.39%, while the proposed method records the lowest at 5.24%. The proposed algorithm also performs optimally in terms of computational efficiency. Additionally, visual comparisons in Fig 15 confirm that the 3D reconstruction of the downsampled point cloud using the proposed method outperforms all other comparative algorithms.

An evaluation of the metrics employed in the analysis of 3D reconstruction results for vegetation point cloud data (see Table 14) reveals the following observations: In terms of geometric fidelity, the MLS algorithm yielded the largest APtSD, measuring at 1.51 mm, while the Voxel Grid algorithm demonstrated the largest MPtSD, measuring at 25.78 mm. Consistent with the building point cloud data, the proposed method attains the smallest APtSD and MPtSD, at 0.86 mm and 16.29 mm respectively. With regard to structural integrity, the MLS algorithm once again exhibited the highest fracture rate, at approximately 32.77%. The proposed method achieved the lowest fracture rate, at 20.77%. The Voxel Grid algorithm demonstrates optimal levels of computational efficiency. Furthermore, visual comparisons in Fig 16 demonstrate that the 3D reconstruction quality of the downsampled point cloud via the proposed method is superior to all other comparative algorithms.

**Table 14:** Evaluation of 3D reconstruction results (Dataset 2).

Method\indicator	Geometric fidelity(mm)		Structural integrity	Computational efficiency	
	APtSD	MPtSD	Breakage rate (%)	reconstruction time(s)	Memory occupancy (MB)
Tensor Voting	1.50	24.47	25.30	9.66	44.00
MLS	1.51	23.98	32.77	10.33	60.00
MST	1.04	18.84	26.63	11.00	59.33
Voxel Grid	1.19	25.78	22.16	6.24	44.00
RGNF	1.04	17.42	23.22	7.51	43.55
Proposed Method	0.86	16.29	20.77	8.24	42.66

As indicated by the evaluation metrics for 3D reconstruction results of road point cloud data (Table 16): In terms of geometric fidelity, the Tensor Voting algorithm has the largest APtSD at 0.97 mm and the largest MPtSD at 33.80 mm. In comparison, the proposed method achieves the smallest MPtSD at 16.29 mm. Regarding structural integrity, the Tensor Voting algorithm records the highest fracture rate at approximately 47.44%, while the proposed method has the lowest at 13.22%. The proposed algorithm also demonstrates optimal computational efficiency. Visual comparisons in Fig 17 further confirm that the 3D reconstruction of the downsampled point cloud using the proposed method outperforms all other comparative algorithms.

Experimental data from Tables 11, 13, and 15 indicate that the proposed method generates smaller downsampled point clouds for most test samples, demonstrating a more universal advantage in data compression. Evaluation results of 3D reconstruction (Tables 12, 14, and 16) are summarized below. In terms of geometric fidelity, the proposed method achieves the smallest APtSD and MPtSD for both building and vegetation ground objects (buildings: 1.97 mm and 10.34 mm; vegetation: 0.86 mm and 16.29 mm). For structural integrity, our method yields the lowest fracture ratios across all three ground object types (5.24%, 20.77%, and 13.22%, respectively). In terms of computational efficiency, the proposed method delivers optimal performance for both building and road point clouds. Combined with the visual reconstruction results in Figs 15, 16, and 17, our method consistently outperforms comparative algorithms including Tensor Voting and MLS in the 3D reconstruction quality of downsampled point clouds.

**Table 15:** Number of points in lightweighted point clouds by different algorithms (Roads).

Data\algorithm	Point Count	Tensor Voting	MLS	MST	Voxel Grid	RGNF	Proposed Method
Road1	28654	27000	20321	26587	24743	25687	20708
Road2	14256	13000	8650	12364	9706	12689	10564
Road3	20587	20000	19421	22654	18515	23421	19753

**Table 16:** Evaluation of 3D reconstruction results (Dataset 3).

Method\indicator	Geometric fidelity(mm)		Structural integrity	Computational efficiency	
	APtSD	MPtSD	Breakage rate (%)	reconstruction time(s)	Memory occupancy (MB)
Tensor Voting	0.97	33.80	47.44	5.00	49.00
MLS	0.64	28.21	29.16	2.66	35.00
MST	0.41	22.31	15.33	2.33	34.33
Voxel Grid	0.59	32.16	15.64	2.00	35.33
RGNF	0.35	21.73	14.51	2.33	34.00
Proposed Method	0.42	19.15	13.22	1.55	17.66

### 3.5. Parameter sensitivity evaluation

To quantitatively evaluate the robustness of the proposed framework to key parameters, we conducted a sensitivity analysis for two core parameters: 1) the window half-width parameter M of the adaptive sliding window; 2) the dynamic threshold coefficient α for AABB weight entropy-driven partitioning.

#### 3.5.1. Sensitivity analysis of window half-width *M.*

We tested the effects of the window half-width M, on the algorithm’s noise removal rate and edge preservation ratio, using building point cloud data (Dataset 1) with M set to a series of values ranging from 10 to 30. The results are summarized in Table 17.

**Table 17:** Experimental results of window half-width M sensitivity analysis.

Value	Noise Removal Rate (%)	Edge Retention Rate (%)
10	85.1	83.8
15	87.6	83.1
20	88.9	82.5
25	89.4	80.3
30	89.5	77.6

We observe from Table 17 that as M increases from 10 to 20, the noise removal rate rises significantly from 85.1% to 88.9%, while the edge retention rate decreases slightly from 83.8% to 82.5%. This indicates that within this range, the algorithm effectively removes noise while well preserving detailed geometric features. When M exceeds 25, the noise removal rate plateaus at approximately 89.5%, yet the edge retention rate drops markedly to below 78%. This phenomenon arises because an overlarge window causes over-smoothing of sharp geometric features.

#### 3.5.2. Sensitivity analysis of entropy threshold coefficient α.

For vegetation point cloud data (Dataset 2), we tested the effects of the coefficient α on skeleton extraction time and topology integrity, with α set to four values: 0.6, 0.8, 1.0, and 1.2. The results are shown in Table 18.

**Table 18:** Experimental results of entropy threshold coefficient α sensitivity analysis.

Value	Topology integrity	Processing Time (s)
0.6	0.94	9.10
0.8	0.93	5.20
1.0	0.91	4.60
1.2	0.89	4.10

With α=0.8, the optimal value selected in this study, the algorithm achieves the optimal balance between topology integrity (0.93) and processing time (5.2 s). When α is reduced to 0.6, topology integrity increases slightly to 0.94, but processing time rises sharply to 9.1 s due to excessive subdivision caused by the smaller threshold. When α is increased to 1.2, processing time decreases to 4.1 s, yet topology integrity drops significantly to 0.89. This performance degradation arises from the excessively coarse partitioning granularity, which leads to the loss of branch information.

### 3.6. Ablation studies

To quantitatively evaluate the independent contributions and synergistic effects of each component in the proposed multi-module coupled framework, we strictly follow the standard ablation study paradigm in computer vision. We design a three-layer progressive experimental system consisting of single-module ablation, combined validation, and scenario-specific overall performance evaluation. All experiments are conducted on three typical ground objects: buildings, vegetation, and roads. All comparative experiments use identical hardware environments and data preprocessing pipelines, with consistent experimental parameters across all runs.

#### 3.6.1. Single-module independent contribution ablation studies.

This experiment uses a traditional point cloud lightweight baseline method (voxel grid downsampling + standard MAT skeleton extraction + PCA normal estimation) as the reference. We sequentially add each of the four proposed modules individually: Module A: multi-scale adaptive sliding window polynomial fitting filter; Module B: entropy-driven adaptive AABB recursive spatial partitioning; Module C: curvature-weighted enhanced medial axis transform (MAT) algorithm; Module D: dihedral angle constraint-based normal vector correction. Three widely accepted evaluation metrics in 3D reconstruction are selected: average point-to-surface distance, topological integrity, and processing time per million points. The quantitative results are presented in Table 19.

**Table 19:** Results of single-module independent contribution ablation studies.

Model	APtSD (mm)	Topology integrity	Million Point Processing Time (s)
Baseline	4.27	0.76	6.81
Baseline +A	3.18	0.81	7.53
Baseline +B	3.89	0.82	5.11
Baseline +C	2.95	0.90	5.87
Baseline +D	4.17	0.78	7.05

The results (Table 19) demonstrate that adding Module A alone reduces the APtSD by 25.5% and improves topological integrity by 6.6%, confirming that high-quality denoising is the foundation for enhancing geometric fidelity. The processing time increases by 10.6%, primarily due to the additional computational overhead of multi-resolution weight calculation and two-level window optimization. Adding Module B alone reduces processing time by 25.0% and improves topological integrity by 7.9%, verifying the advantage of adaptive spatial partitioning in balancing computational efficiency and feature preservation. Conversely, the improvement in geometric fidelity is relatively limited (8.9%), indicating that its primary role is to optimize the computational pipeline rather than directly enhance geometric features. Adding Module C alone achieves the largest improvements in both geometric fidelity (30.9%) and topological integrity (18.4%), highlighting that skeleton extraction is a critical step determining the quality of point cloud lightweighting. Adding Module D alone reduces the APtSD by 2.3%, improves topological integrity by 2.6%, and increases processing time by 3.5%, demonstrating that normal correction provides a small but consistent improvement in final reconstruction accuracy.

#### 3.6.2. Module combination synergistic effect ablation studies.

To verify that the proposed coupled framework is not a simple superposition of modules but exhibits significant synergistic enhancement effects, we design a progressive module combination scheme. We further replace the original normal estimation module of the baseline method with Module D (dihedral angle constraint-based normal vector correction). The quantitative results are presented in Table 20.

**Table 20:** Results of module combination synergistic effect ablation studies.

Model	APtSD (mm)	Topology integrity	Million Point Processing Time (s)
Baseline	4.27	0.76	6.81
Baseline +A + B	2.56	0.85	6.31
Baseline +A + B + C	1.12	0.91	5.68
Baseline +A + B + C + D	1.08	0.93	5.20

We observe that combining Modules A and B reduces the APtSD by 40% and processing time by 7.3%. This occurs because noise removal by the filter enables the entropy-driven partitioning to more accurately identify feature-dense regions, avoiding excessive computation in uniform areas and thus offsetting part of the time overhead introduced by the filtering module. Adding Module C on the basis of A + B further reduces the APtSD by 56.2% and improves topological integrity by 7.1%. This confirms that after adaptive spatial partitioning divides the point cloud into uniform sub-regions, the enhanced MAT algorithm can more efficiently compute the distance field and identify local maxima, reducing cross-region topological fractures. The synergistic effect is particularly pronounced in complex topological scenarios such as vegetation and roads. The full proposed method further reduces the APtSD by 3.6%, improves topological integrity by 2.2%, and decreases processing time by an additional 8.5%. This indicates that normal correction optimizes the normal field at sharp features, slightly improving geometric accuracy while accelerating the surface fitting process in subsequent 3D reconstruction. Compared with the baseline method, the full proposed method reduces the overall average point-to-surface distance by 74.7%, improves topological integrity by 22.4%, and decreases processing time by 23.6%.

#### 3.6.3. Overall results of scenario-specific ablation studies.

To verify the universality of the proposed method across different ground object types, we conduct complete ablation studies in three typical scenarios: buildings, vegetation, and roads. The quantitative results are presented in Table 21.

**Table 21:** Overall results of scenario-specific ablation studies (APtSD/mm).

Ground Object Type	Baseline	Baseline +A	Baseline +A + B	Baseline +A + B + C	Baseline +A + B + C + D
Buildings	4.25	3.84	2.68	2.05	1.97
Vegetation	5.12	3.15	2.82	0.88	0.86
Roads	3.44	2.55	2.18	0.43	0.42
Average	4.27	3.18	2.56	1.12	1.08

The overall results of scenario-specific ablation studies (Table 21) show that the proposed method achieves significant reconstruction accuracy improvements in all three scenarios. For buildings with regular structures, the proposed method reduces the APtSD by 53.6%, with Module C contributing the most to topological integrity. Module A reduces the APtSD from 4.25 mm to 3.84 mm through denoising, laying the foundation for subsequent feature extraction. For roads with linear structures, the proposed framework reduces the APtSD by 87.8%, with Module B significantly optimizing computational efficiency through adaptive spatial partitioning. For vegetation with complex fractal structures, Module A reduces the APtSD from 5.12 mm to 3.15 mm, removing significant high-frequency noise interference. Adding Module B’s adaptive spatial partitioning on the basis of Module A’s denoising further reduces the APtSD to 2.82 mm. It also reorganizes the point cloud into structured sub-regions, significantly improving the topological accuracy of subsequent skeleton extraction and laying the foundation for the substantial accuracy improvement achieved by Module C. Module C accurately extracts the fine branch skeletons of vegetation through curvature-weighted sampling, sharply reducing the APtSD to 0.88 mm. Finally, Module D optimizes the accuracy to 0.86 mm through normal correction, fully demonstrating the synergistic advantages of deep multi-module coupling.

Through the systematic ablation studies described above, we draw the following conclusions. All three innovative modules proposed herein make significant contributions to improving point cloud lightweighting performance. The enhanced MAT algorithm is a critical component determining topological integrity, adaptive filtering forms the foundation for improving geometric fidelity, and entropy-driven partitioning is the key to achieving efficient computation. Significant synergistic enhancement effects exist between modules. The performance of the full-process coupled framework far exceeds the linear superposition of the individual modules’ effects, verifying the rationality of the proposed method’s design. The proposed method achieves excellent performance in all three typical scenarios of buildings, vegetation, and roads, demonstrating its universality in lightweight processing of complex urban ground object point clouds.

### 3.7. Generalisation validation

**Fig 19:**
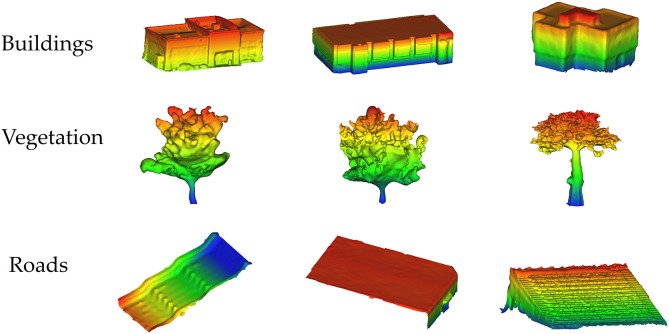
3D reconstruction results on the STPLS3D dataset.

To further verify the cross-scene generalization capability of the proposed algorithm, overcome the scene-specific limitations of single-scene measured data, and fully demonstrate the algorithm’s universal adaptability to heterogeneous-density point clouds acquired from diverse urban scenes and different collection conditions, we conducted generalization validation experiments using the Synthetic and Real-world Point Cloud Dataset for Large-Scale 3D Semantic Segmentation (STPLS3D) [61] dataset. This is a widely used large-scale urban scene benchmark dataset in the international point cloud processing community. The STPLS3D dataset covers diverse urban scenarios, including urban core areas, industrial parks, and urban-rural fringe zones. It contains 16 categories of finely annotated urban scene elements, such as buildings, vegetation, roads, and municipal facilities, with a wide range of point cloud densities, full coverage of noise levels, and a complete gradient of ground object complexity. This validation strictly followed the standard train-test split rules defined for the STPLS3D dataset. Three typical ground object types in the dataset (buildings, vegetation, and roads) were selected for analysis. We used the same algorithm parameter configuration and evaluation index system as the main experiments, and conducted a comprehensive assessment of the algorithm’s generalization performance on the public benchmark dataset across three quantitative dimensions: geometric fidelity, structural integrity, and computational efficiency of 3D reconstruction. Visual experimental results are presented in Fig 19, and the evaluation metrics for 3D reconstruction are summarized in Table 22.

**Table 22:** Evaluation of 3D reconstruction performance (STPLS3D Dataset).

Method\indicator	Geometric fidelity(mm)		Structural integrity	Computational efficiency	
	APtSD	MPtSD	Breakage rate (%)	reconstruction time(s)	Memory occupancy (MB)
Building①	2.05	8.26	7.89	10.58	101.57
Building②	1.92	9.87	5.12	13.86	105.32
Building③	8.51	13.53	4.96	11.24	98.75
Vegetation①	0.92	17.15	21.58	5.65	43.89
Vegetation②	1.83	16.02	20.35	8.12	42.16
Vegetation③	0.80	25.68	19.87	5.89	41.53
Road①	0.45	20.26	10.89	1.68	15.52
Road②	0.41	19.03	13.15	1.52	17.46
Road③	0.39	18.65	17.78	2.45	19.98

Validation results in Table 22 show that the proposed method delivers stable reconstruction performance across all three typical ground object scenarios: buildings, vegetation, and roads. For building scene datasets, with the exception of one sample group, the APtSD stabilizes between 1.92 mm and 2.05 mm. The MPtSD across all samples is constrained within 13.53 mm, and the minimum reconstruction fracture ratio reaches as low as 4.96%. For vegetation scene datasets, the minimum APtSD is 0.80 mm, with a full-sample range of 0.80 mm to 1.83 mm. The reconstruction fracture ratio remains stable between 19.87% and 21.58%, demonstrating the method’s excellent geometry-preserving capability for complex vegetation structures with fine branches and high curvature. For road scene datasets, the APtSD is only 0.39 mm to 0.45 mm, the optimal performance among the three scenarios. The reconstruction fracture ratio is controlled within 10.89% to 17.78%, with the shortest reconstruction time for a single sample group at just 1.52 s and a minimum memory footprint as low as 15.52 MB. These results confirm that the proposed method achieves dual advantages in both accuracy and efficiency for the simplification and reconstruction of banded linear ground objects.

## 4. Conclusions

To address the key challenges of insufficient noise robustness and the difficulty of balancing geometric feature preservation and computational efficiency in complex ground object point cloud simplification, this study proposes a technical framework integrating multi-scale adaptive filtering and entropy-driven partitioning. The key achievements of this work are summarized below:

Point cloud filtering: The proposed adaptive sliding window polynomial fitting filter achieves complementary noise suppression and feature preservation across scales via a multi-resolution weight adjustment mechanism. We designed this method to enhance the processing of sharp feature regions by incorporating a dynamic optimization strategy for two-level feature edge windows. Across building, vegetation, and road point cloud datasets, the proposed method achieves an average noise removal rate of 87.76% and an edge preservation rate of 83.3%. For sharp features such as building edges and road boundaries, it achieves a maximum improvement of 11.37% compared with conventional filtering methods, providing a high-fidelity data foundation for subsequent skeleton extraction and normal estimation.

Skeleton extraction: The enhanced medial axis transform (MAT) algorithm uses curvature-weighted sampling to dynamically adjust skeleton point density. Combined with a dynamic radius neighborhood search, this effectively reduces skeleton false branching and structural fragmentation caused by noise. Experimental results show that the algorithm attains a topology integrity of 0.93 for building point clouds and a branch accuracy of 0.95 for vegetation point clouds, outperforming comparative algorithms including KNN and MST. Furthermore, the efficient topological construction logic enables the algorithm to process million-point-scale data within 5.2 seconds, a 29.7% reduction in processing time compared with the KNN algorithm. This achieves a favorable balance between complex topological structure characterization and computational efficiency.

Spatial partitioning strategy: The hierarchical AABB adaptive recursive partitioning method dynamically adjusts partitioning granularity based on the weight entropy of sub-bounding boxes, optimizing computational resource allocation according to point cloud density heterogeneity. This method reduces memory usage by 32% in sparse regions (e.g., roads) and increases the feature retention rate to 94% in high-density regions (e.g., buildings). It effectively mitigates the resource waste and feature loss issues associated with traditional uniform partitioning, providing structural support for efficient processing of large-scale point clouds.

Normal estimation and 3D reconstruction experiments further validate the comprehensive performance of the proposed method. The mean normal estimation error is reduced to 3.34%, and the computational efficiency for vegetation point clouds reaches 89.14%, significantly outperforming comparative algorithms including Tensor Voting and MST. 3D reconstruction results show that the method can compress the downsampled point cloud size to 50%–60% of the original size, while retaining more than 80% of key geometric features. Specifically, the mean point-to-surface distances for building and vegetation point clouds are as low as 1.97 mm and 0.86 mm, respectively. Reconstruction time and memory usage for building and road scenarios are within reasonable ranges, and the visual fidelity is superior to existing comparison methods. Systematic ablation experiments further clarify the underlying mechanisms of these performance improvements. The results show that the enhanced MAT algorithm makes the largest contribution to topological integrity. Adaptive filtering and entropy-driven partitioning, meanwhile, play key roles in improving geometric fidelity and computational efficiency, respectively. Importantly, significant synergistic effects exist between modules, allowing the fully coupled framework to outperform the linear superposition of individual components across all three typical ground object scenarios. In addition, we completed cross-scene generalization validation of the proposed method using the STPLS3D dataset, a widely recognized benchmark for large-scale urban scenes in the international point cloud processing community. Results show that the proposed method delivers stable performance in geometry preservation and topological structure integrity when processing three typical ground objects (buildings, vegetation, and roads) across diverse urban scenes, fully verifying its cross-scene adaptability and robustness for practical engineering applications.

Compared with deep learning networks focused on feature learning, the proposed method achieves competitive noise removal rates and superior geometric detail preservation across the three ground object datasets via well-defined geometric rules. It offers particularly straightforward deployment value in practical engineering scenarios with limited computational resources and training data. In contrast to existing lightweight hybrid geometry-learning frameworks, the key advance of the proposed method is its shift from heavy reliance on deep learning architectures to in-depth exploitation of the inherent geometric and statistical properties of point clouds. This design ensures the method’s universal adaptability to the intrinsic patterns of point clouds from diverse scenarios.

Despite these promising results, our study has several limitations. Our experimental platform is equipped with an Intel Core i7-10700K CPU (3.8 GHz), 32 GB of RAM, and an NVIDIA RTX 3080 GPU with 10 GB of GPU memory, running on Microsoft Windows 11. The limited GPU memory prevents us from processing large-scale urban point clouds using recent end-to-end deep learning models. Second, our self-collected field-measured dataset only contains coarse-grained annotations for three ground object categories: buildings, vegetation, and roads. Most mainstream deep learning-based point cloud lightweighting methods require fine-grained semantic annotations for model training. Together, these two constraints prevent us from conducting end-to-end full pipeline comparisons with representative deep learning methods such as SampleNet and LSNet. For ultra-large-scale heterogeneous ground object point clouds at the urban scale, the parallel computing load balancing of the adaptive AABB partitioning algorithm requires improvement. Under intense noise interference, the iterative convergence efficiency of the normal vector correction algorithm can be further optimized. To address these limitations, future research will focus on three priority directions. First, we will continuously benchmark against state-of-the-art mainstream methods in point cloud simplification from the past 3–5 years, conduct multi-dimensional performance comparisons, and implement targeted iterative optimization of the algorithm to further refine the technical framework and overall performance. Second, we will explore a hybrid-driven mechanism integrating deep learning and geometric rules, using neural network models to predict optimal partitioning parameters to significantly improve the processing efficiency of ultra-large-scale point cloud data. Finally, we will develop acceleration strategies for normal correction based on curvature feature abrupt change detection. By optimizing the iterative process, we will reduce the method’s computational complexity under extreme noise conditions, to further enhance its universal applicability in complex practical engineering scenarios.

## Supporting information

SI File Supplementary technical details of algorithm parameters. This supplementary file contains parameter initialization principles, grid search optimization basis, cross-dataset validation results, noise sensitivity tests, and three standardized supplementary tables (Table S1–S3) for the proposed point cloud lightweight method.
(DOCX)
