# Supplementary material for: Robust point cloud lightweighting with multi-scale adaptive filtering and entropy-driven subdivision
Source: PLoS One. 2026 Jul 24;21(7):e0353953. doi: 10.1371/journal.pone.0353953 (PMC13399364; doi:10.1371/journal.pone.0353953)
Supplement: SI File — This supplementary file contains parameter initialization principles, grid search optimization basis, cross-dataset validation results, noise sensitivity tests, and three standardized supplementary tables (Table S1–S3) for the proposed point cloud lightweight method. (DOCX) [file pone.0353953.s001.docx]

# Supplementary Information

# Corresponding Article

*Robust Point Cloud* *Lightweighting with Multi-Scale Adaptive Filtering and Entropy-Driven Subdivision*

# Manuscript ID

PONE-D-26-04255

# Authors

Weibo Zeng ^1,3^, Xinyu Gao ^1^, Qi Lu ^1^, Ning Zhu ^1^, Mengchan Li ^2^ and Wenjing Cai ^2^

# Preface

This Supplementary Information (SI) provides key technical details for our robust point cloud lightweighting method based on multi-scale adaptive filtering and entropy-driven subdivision. It covers core parameter initialization rules, rationale for parameter value selection, optimization workflows, scenario-specific parameter adaptation strategies, and standardized computing environment specifications.

All content aligns completely with the methods and experiments described in the main manuscript. We have validated all parameters and configuration workflows across datasets for three typical urban ground objects: buildings, vegetation, and roads. This parameter configuration is fully reproducible, allowing other researchers to replicate our method.

# S1 Core Principles of Parameter Initialization and Optimization Validation Methods

## S1.1 Core Principles of Parameter Initialization

All core parameter initialization and value selection herein follow three core principles, to ensure that parameter settings have clear physical meanings, rigorous theoretical basis, and scenario adaptability:

1. Principle of Prioritizing Physical Meaning: All parameters correspond to specific geometric features, spatial distribution characteristics, or algorithm constraints in the point cloud processing workflow. No empirically tuned parameters without clear physical meaning are included. Parameter values are directly correlated with the average point spacing, local curvature, and spatial distribution density of the input point cloud.

2. Principle of Balancing Accuracy and Efficiency: Parameter optimization targets the dual balance of noise suppression versus detail preservation, and topological fidelity versus lightweight efficiency. Priority is given to parameter values that achieve the optimal balance across key evaluation metrics, to avoid overall performance imbalance caused by optimizing for a single metric alone.

3. Principle of Scenario Universality: The initialization range and optimal values of all parameters are cross-validated across three typical urban ground objects: buildings, vegetation, and roads. This ensures that the parameter configuration has stable adaptability for point cloud data with different geometric features and density distributions.

## S1.2 Parameter Optimization and Validation Methods

We first determine the initialization range and step size for each parameter based on general experience in the point cloud processing field and theoretical constraints of the algorithm. A control variable method is adopted, where all other parameters are fixed while only the value of the single target parameter is adjusted. We then record changes in the module’s core evaluation metrics under different values of the target parameter.

Multiple sets of grid search experiments are conducted, with the weighted comprehensive score of multiple metrics as the evaluation criterion. The parameter value that achieves the optimal overall performance of the module is selected as the final value. Cross-validation of the optimal parameter configuration is performed on the three types of ground object datasets, to ensure stable performance of the parameters across different scenarios.

# S2 Value Selection and Optimization Basis of Core Parameters

The method herein consists of four core modules: adaptive sliding window polynomial fitting filtering, entropy-driven AABB adaptive recursive subdivision, curvature-weighted medial axis transforms skeleton extraction, and normal estimation and correction. The initialization rules, specific values, corresponding formulas in the main manuscript, and optimization basis of key parameters for each module are listed in Table S1.

All parameters have been verified through the full workflow in the experimental scene of a campus built-up area, with favorable scenario adaptability and robustness.

**Table S1** Values and Basis of Core Key Parameters

| Core Module | Parameter | Value | Equation | Optimization Basis |
| --- | --- | --- | --- | --- |
| Adaptive Sliding Window Polynomial Fitting Filtering | Half-width of filtering window $M$ | 20 | Eq. (4) | When $M<15$, the window coverage is insufficient, resulting in poor suppression effect on non-uniform noise. When $M>25$, the window causes over-smoothing and severe loss of sharp edge features. The optimal balance between noise removal rate and edge preservation is achieved at $M=20$. |
|  | Polynomial order $p$ | 2 | Eq. (4) | 1st-order linear fitting cannot adapt to the features of complex curved surfaces, while 3rd-order cubic fitting is prone to overfitting and causes a sharp increase in computational cost. 2nd-order quadratic fitting achieves the optimal balance among fitting accuracy, noise resistance, and computational efficiency. |
|  | Minimal constant for coarse-scale weight calculation $\epsilon_{f}$ | 10^−8^ | Eq. (10) | It prevents the denominator from being zero without affecting the calculation accuracy of coarse-scale weights, and adapts to the numerical distribution characteristics of the local variance of the point cloud. |
|  | Fusion weight of the secondary window $\alpha_{f}$ | 0.6~0.8 | Eq. (12) | When $\alpha_{f}<0.5$, the proportion of the secondary window is excessively high, leading to insufficient noise suppression in planar areas. When $\alpha_{f}>0.9$, the proportion of the main window is excessively high, resulting in poor preservation of sharp features. The range of 0.6–0.8 can balance noise suppression and edge feature preservation. |
| Entropy-Driven AABB Adaptive Recursive Subdivision | Entropy threshold coefficient $\alpha$ | 0.8 | Eq. (22) | When $\alpha<0.6$, the segmentation threshold is too small, resulting in over-subdivision of sub-bounding boxes and a sharp increase in computational cost. When $\alpha>1.0$, the segmentation threshold is too large, leading to insufficient subdivision and loss of features in high-density areas. A value of 0.8 achieves the optimal balance between topological integrity and processing efficiency. |
|  | Entropy standard deviation weight coefficient $\beta$ | 0.4 | Eq. (22) | It adapts to the distribution characteristics of point cloud entropy values in urban scenes, dynamically balances the influence of average entropy and local entropy fluctuation on the segmentation threshold, and achieves the optimal scenario adaptability. |
|  | Curvature sampling weight coefficient $w_{k}$ | 0.65 | Eq. (24) | hen $w_{k}<0.5$, the sampling density in high-curvature areas is insufficient, resulting in the loss of fine branch details. When $w_{k}>0.8$, sampling redundancy occurs in high-curvature areas, reducing the lightweight effect. A value of 0.65 balances the lightweight efficiency in flat areas and detail preservation of complex structures. |
| Curvature-Weighted Medial Axis Transform Skeleton Extraction | Neighborhood search radius $\epsilon$ | 0.1 | Eq. (23) | When $\epsilon$<0.05 m, the neighborhood coverage is insufficient, and the distance field calculation is susceptible to noise interference. When $\epsilon$>0.15 m, the neighborhood range is excessively large, and local detail features are smoothed. A value of 0.10 m adapts to the average point spacing of the point cloud in the experimental scene, and balances the calculation accuracy of the distance field and noise resistance. |
|  | Normal angle threshold for medial point connection $\theta_{threshold}$ | 0.90 | Eq. (29) | When $\theta_{threshold}$<0.85, the normal consistency constraint is too loose, and false branches are easily generated. When $\theta_{threshold}$>0.95, the constraint is too strict, and skeleton fracture is prone to occur. A value of 0.90 balances the topological connectivity and structural regularity of the skeleton. |
| Normal Estimation | Loose threshold coefficient for sharp feature screening $\alpha_{n}$ | 1.2 | Eq. (27) | It adapts to the distribution characteristics of topographic relief intensity, realizes loose screening in high-relief areas, fully retains sharp feature points, and avoids feature missing detection. |
|  | Strict threshold coefficient for sharp feature screening $\beta_{n}$ | 0.8 | Eq. (28) | It realizes strict screening in flat areas, reduces the false detection rate of pseudo-feature points, and ensures the stability of normal estimation in planar areas. |
|  | Normal difference tolerance threshold | 5°~15° | Eq. (29) | It is set to 5°~8° for flat areas to improve the consistency of the normal field, and 10°~15° for high-curvature sharp feature areas to preserve the normal mutation characteristics at sharp features. This setting adapts to the geometric features of different ground objects. |

# S3 Stepwise Calculation Workflow of Semantic-Geometric Coupled Weights

The full-workflow parameter initialization of the method herein takes the average point spacing $\overline{d}$ of the input point cloud as the fundamental benchmark. It follows three core workflows: basic parameter initialization, main module parameter configuration, and parameter validity verification.

The full-workflow configuration is completed in combination with the optimal parameter values in Table S1. The operation steps are specific and reproducible, and the final output parameter configuration can be directly applied to the full-workflow point cloud lightweight processing of the method herein.

## S3.1 Basic Parameter Initialization

Based on the input point cloud, we calculate the global characteristic parameters of the point cloud and complete the initialization of the basic algorithm parameters, which provides a benchmark for the parameter configuration of each module:

1. Calculate the average point spacing $\overline{d}$ of the input point cloud, which serves as the initialization benchmark for neighborhood-related parameters.

2. Statistically analyze the elevation distribution and local curvature distribution characteristics of the point cloud, and calculate the mean $\mu_{R}$ and standard deviation $\sigma_{R}$ of the topographic relief intensity. These metrics provide the calculation basis for the sharp feature screening threshold.

3. Statistically obtain the overall bounding box range of the point cloud and the total number of points $N$, to determine the benchmark for the initial level and termination conditions of AABB recursive subdivision.

## S3.2 Basic Parameter Initialization

Based on the optimal parameter values in Table S1, we complete the standardized parameter configuration of the four core modules. The stepwise calculation and configuration workflow of parameters for each module are described below.

1. Parameter Configuration of the Filtering Module

We set the polynomial order $p$, and select three commonly used levels in practical applications: 1 (linear fitting), 2 (quadratic fitting), and 3 (cubic fitting). For each order $p$, the window size L is set to 7 groups of candidate values: $p+2,p+3,p+4,p+5,2p+3,2p+4,2p+5$. Quantitative evaluation is performed using three metrics: noise removal rate, edge preservation degree, and fitting error. The results demonstrate that the optimal comprehensive performance is achieved when $L=\left[ 2p+3 \right]$. The sliding window size is calculated according to this formula, where the polynomial order $p=2$, corresponding to a window half-width $M=20$. We set the secondary window width $L2=M+1$, the fusion weight $\alpha\in[0.6,0.8]$, and the minimal constant for coarse-scale weight calculation $\epsilon$=10^−8^, completing the parameter configuration of the filtering module.

2. Parameter Configuration of the AABB Subdivision Module

We set the dynamic segmentation threshold coefficients $\alpha$=0.8 and $\beta$ =0.4 according to Eq. (22), along with the curvature sampling weight coefficient $w_{k}$=0.65. The minimum level $h_{min}$=2 and maximum level $h_{max}$=8 of recursive subdivision is set, completing the parameter configuration of the spatial subdivision module.

3. Parameter Configuration of the Skeleton Extraction Module

We set the neighborhood search radius $\epsilon$=0.10 m according to the empirical formula $\epsilon$∈ [3$\overline{d}$,8$\overline{d}$]. The local maximum search radius is set to $r_{max}$=1.5×ϵ=0.15m m, and the normal angle threshold for medial point connection is set to $\theta_{threshold}$=0.90. This completes the parameter configuration of the skeleton extraction module.

4. Parameter Configuration of the Normal Estimation and Correction Module

The sharp feature screening thresholds are calculated according to Eq. (27) and Eq. (28), with coefficients $\alpha$=1.2 and $\beta$ =0.8. The normal difference tolerance threshold is set to 5°~8° for flat areas and 10°~15° for high-curvature areas, completing the parameter configuration of the normal estimation module.

## S3.3 Basic Parameter Initialization

After completing the parameter configuration, two verification steps are performed to ensure the validity and rationality of the parameter settings:

1. Single-module performance verification: Control experiments are conducted separately for each module to verify whether the core evaluation metrics reach the performance level reported in the main manuscript. If the metric deviation exceeds 5%, parameter optimization is re-conducted.

2. Full-workflow stability verification: Full-workflow point cloud lightweight processing for the three types of ground objects is performed using the configured parameters. We verify the algorithm’s operational stability, result accuracy, and computational efficiency, to ensure favorable adaptability of the parameter configuration throughout the full workflow.

# S4 Cross-Dataset Parameter Consistency Verification and Data Characteristic Sensitivity Analysis

All core algorithm parameters were kept identical between our self-collected Chuzhou University Measured Dataset and the public STPLS3D Benchmark Dataset. We did not perform any dataset-specific parameter tuning for either dataset. The universality of these parameters arises from their initialization mechanism, which is based on the inherent geometric properties of point clouds rather than empirical fitting to specific scenarios.

## S4.1 Cross-Dataset Performance Consistency Verification

To verify the performance stability under unified parameter configuration, we compared the core metrics of the proposed method on three typical ground objects across the two datasets. The results are presented in Table S2.

**Table S2**. Cross-Dataset Performance Metric Comparison

| Ground Object Type | Evaluation Metric | Measured dataset | STPLS3D dataset |
| --- | --- | --- | --- |
| Buildings | Noise Removal Rate (%) | 89.2 | 88.7 |
|  | Topological Completeness | 0.93 | 0.92 |
|  | APtSD | 1.97 | 2.00 |
| Vegetation | Noise Removal Rate (%) | 88.4 | 87.9 |
|  | Topological Completeness | 0.95 | 0.94 |
|  | APtSD | 0.86 | 0.89 |
| Roads | Noise Removal Rate (%) | 85.7 | 85.2 |
|  | Topological Completeness | 0.95 | 0.94 |
|  | APtSD | 0.42 | 0.41 |

These results show that under identical parameter settings, all core metrics on the STPLS3D dataset deviate by less than 3.5% from those on our Measured Dataset. This validates both the cross-scene universality of our parameter configuration and the generalization ability of our method. The minor performance differences primarily result from differences in data acquisition devices and environmental conditions between the two datasets.

## S4.2 Sensitivity to Noise Level Variations

We added Gaussian noise with different intensities (σ = 0.05, 0.1, 0.15, and 0.2 m) to the original point cloud data to test the anti-noise performance of the algorithm. All experiments were conducted using the parameter configuration shown in Table S1, and the results are summarized in Table S3.

**Table S3**. Performance Metrics Under Different Noise Levels

| Gaussian Noise Standard Deviation (m) | Noise robustness | APtSD/mm | Breakage rate (%) |
| --- | --- | --- | --- |
| 0.05 | 0.96 | 0.92 | 2.1 |
| 0.10 | 0.92 | 1.08 | 3.5 |
| 0.15 | 0.87 | 1.34 | 5.8 |
| 0.20 | 0.81 | 1.67 | 8.2 |

When the noise intensity $\sigma\leq0.1$ m, the Noise Robustness Score (NRS) of the algorithm remains above 0.92, the APtSD increases by less than 20%, and the breakage rate is below 4%. Even under extreme noise conditions with σ = 0.2 m, the algorithm still maintains an NRS of 0.81. This demonstrates its excellent stability in complex engineering scenarios with severe noise interference.
